# Regulation of terminal hypertrophic chondrocyte differentiation in *Prmt5* mutant mice modeling infantile idiopathic scoliosis

**DOI:** 10.1242/dmm.041251

**Published:** 2019-12-17

**Authors:** Zhaoyang Liu, Janani Ramachandran, Steven A. Vokes, Ryan S. Gray

**Affiliations:** 1 Department of Pediatrics, Dell Pediatric Research Institute, 1400 Barbara Jordan Blvd, The University of Texas at Austin, Dell Medical School, Austin, TX 78723, USA; 2 Department of Molecular Biosciences, 2500 Speedway, The University of Texas at Austin, Austin, TX 78712, USA; 3 Department of Nutritional Sciences, 200 W 24th Street, The University of Texas at Austin, Austin, TX 78712, USA

**Keywords:** Infantile scoliosis, PRMT5, Chondrocyte terminal differentiation, Endochondral ossification

## Abstract

Idiopathic scoliosis (IS) is the most common type of musculoskeletal defect affecting children worldwide, and is classified by age of onset, location and degree of spine curvature. Although rare, IS with onset during infancy is the more severe and rapidly progressive form of the disease, associated with increased mortality due to significant respiratory compromise. The pathophysiology of IS, in particular for infantile IS, remains elusive. Here, we demonstrate the role of PRMT5 in the infantile IS phenotype in mouse. Conditional genetic ablation of PRMT5 in osteochondral progenitors results in impaired terminal hypertrophic chondrocyte differentiation and asymmetric defects of endochondral bone formation in the perinatal spine. Analysis of these several markers of endochondral ossification revealed increased type X collagen (COLX) and *Ihh* expression, coupled with a dramatic reduction in *Mmp13* and RUNX2 expression, in the vertebral growth plate and in regions of the intervertebral disc in the *Prmt5* conditional mutant mice. We also demonstrate that PRMT5 has a continuous role in the intervertebral disc and vertebral growth plate in adult mice. Altogether, our results establish PRMT5 as a critical promoter of terminal hypertrophic chondrocyte differentiation and endochondral bone formation during spine development and homeostasis.

This article has an associated First Person interview with the first author of the paper.

## Abstract (precis)

**Summary:** Loss of *Prmt5* in osteochondral progenitors impairs terminal hypertrophic chondrocyte differentiation, leading to defects in endochondral bone formation and models infantile idiopathic scoliosis in mouse.

## INTRODUCTION

Idiopathic scoliosis (IS) is the most common pediatric spinal deformity, characterized by a lateral curvature of the spine >10° and affecting ∼3% of children worldwide (Wise et al., 2008). Clinical subclassifications of IS are based on age of presentation at infantile (birth to 3 years), juvenile (age 3 to 11 years) and adolescent (11 years and older) ages (Giampietro et al., 2003). Infantile IS develops rapidly and can lead to significant respiratory compromise, increased morbidity and mortality (Cahill and Samdani, 2012; Davies and Reid, 1970; Pehrsson et al., 1992; Roye, 2018). Therefore, there is a strong need for early diagnosis and rational therapies that might halt or ameliorate the pathogenesis of infantile IS.

Sibling risk studies report that 11.5-19% of siblings share risk for spine curvatures >10° (Riseborough and Wynne-Davies, 1973; Rogala et al., 1978). Despite this strong genetic signal, the etiology of IS remains poorly understood. However, recent advances to understand the genetic causality and candidate loci associated with the disease are promising. For instance, large-scale genome-wide association studies also implicated several candidate loci associated with IS, including associations with *GPR126* (*ADGRG6*), *LBX1*, *CHL1* and *SOX9* genes (Guo et al., 2016; Kou et al., 2013; Sharma et al., 2011; Takahashi et al., 2011). Scoliosis is a common phenotype in mutant mouse models that disrupt genes involved with the development and homeostasis of cartilages and connective tissues, including *Adgrg6/Gpr126* (Karner et al., 2015), *Sox9* (Henry et al., 2012), *Shp2* (*Ptpn11*) (Kim et al., 2013), *Gdf5/6* (Settle et al., 2003) and *Fgf3* (Gao et al., 2015). The convergence of human genetic data and mutant models of scoliosis in mouse is indicative of a potential relationship between the physiology of cartilaginous tissues of the spine and the pathogenesis of IS (Liu and Gray, 2018).

The formation of the bony vertebral bodies in mammals proceeds via a process of endochondral ossification of the cartilaginous anlagen flanking the notochord during embryonic development. During postnatal development, the vertebral bodies continue to grow and mature via the proliferation and differentiation of the vertebral growth plate chondrocytes (Smith et al., 2011). The fusion of the growth plate typically occurs during adolescence in humans but persists in adult mice (Börjesson et al., 2012). The intervertebral disc (IVD), connecting the vertebral bodies of the spine, is largely composed of cartilaginous tissues. The nucleus pulposus is the innermost gel-like center of the IVD and is surrounded by numerous fibrocartilaginous lamellar layers collectively known as the annulus fibrosus. The cartilaginous endplate tissue of the IVD is physically connected to the vertebral growth plate and bony vertebrae.

Endochondral ossification is the process by which chondrocytes are replaced by bone. Although this process has largely been studied in the long bone, the mechanisms are comparable in the vertebral growth plate (Karsenty et al., 2009; Long and Ornitz, 2013). This process is characterized by the succession of signaling that controls differentiation of proliferative, prehypertrophic, hypertrophic and terminal hypertrophic chondrocytes (Long and Ornitz, 2013). Endochondral ossification of a vertebra begins within the center of the cartilaginous anlagen of the vertebral body. Cells within this center of ossification exit the cell cycle and initiate hypertrophy, which is marked by the secretion of type X collagen (COLX; encoded by *Col10a1*). Subsequently, matrix metalloproteinase 13 (*Mmp13*) is expressed in these cells, which is critical for terminal differentiation of hypertrophic chondrocytes and endochondral bone formation (Inada et al., 2004). Mechanistically, MMP13 aids in the degradation of the COLX-rich extracellular matrix surrounding the hypertrophic chondrocytes, fostering the invasion of blood vessels attracted by expression of vascular endothelial growth factors (VEGFs) and ultimately promoting the infiltration of bone-forming osteoblasts (Karsenty et al., 2009; Long and Ornitz, 2013). Many of these hypertrophic chondrocytes undergo apoptosis, clearing a way for the elaboration of the bony matrix; however, recent studies demonstrate that a subset of these hypertrophic chondrocytes transdifferentiates into osteoblasts, directly contributing to bone formation (Jing et al., 2015; Park et al., 2015; Zhou et al., 2014).

Chondrocyte maturation and terminal chondrocyte differentiation is tightly regulated by a series of growth factors and transcriptional regulators. For example, the transcriptional regulator SRY-related high-mobility group box 9 (SOX9) is required for chondrocyte proliferation and to prevent precocious hypertrophic differentiation (Akiyama et al., 2002; Karsenty et al., 2009), whereas the transcription factor runt-related transcription factor 2 (RUNX2), directly regulates *Col10a1*, *Mmp13* and *Vegfa* expression (Hess et al., 2001; Li et al., 2011; Takahashi et al., 2017; Zelzer et al., 2001), and is an important driver of hypertrophic chondrocyte differentiation and endochondral ossification (Komori, 2010a,b, 2018). Finally, Indian hedgehog (IHH) signaling plays a crucial role in chondrocyte proliferation and hypertrophic differentiation via PTHrP (Pthlh)-dependent and independent processes (Amano et al., 2014; Long and Ornitz, 2013; Mak et al., 2008). During postnatal development, chondrocytes continue to regulate spine homeostasis by maintaining a dynamic balance between the synthesis and degradation of extracellular matrix components in the cartilaginous tissues of the spine. These processes are regulated in part by key transcriptional regulators, including SOX9 (Henry et al., 2012) and RUNX2 (Alvarez-Garcia et al., 2018; Liao et al., 2019).

The protein arginine methyltransferase 5 (PRMT5) is a critical factor involved in the establishment of chondrocyte progenitors in embryonic limb buds (Norrie et al., 2016), resulting in precocious *Sox9* expression followed by widespread apoptosis and loss of the cartilage template and skeletal element in the limb (Norrie et al., 2016). Mechanistically, PRMT5 is a key enzyme for symmetrical demethylation of a diverse array of molecular targets including histones H3R8, H3R2 and H4R3 (Bedford and Clarke, 2009; Pal et al., 2004; Stopa et al., 2015; Zhao et al., 2009), and small nuclear ribonucleoproteins (Chari et al., 2008; Meister and Fischer, 2002; Stopa et al., 2015), which can affect gene expression and splicing of mRNAs, respectively. PRMT5 has also been shown to regulate the dimethylation of SOX9 protein, increasing its half-life (Sun et al., 2019). Given the range of potential molecular substrates for PRMT5, it is not surprising that this enzyme plays a role in a number of tissue-specific differentiation pathways, including keratinocyte, muscle, nerve cell and lung differentiation (Calabretta et al., 2018; Chittka et al., 2012; Dacwag et al., 2009; Kanade and Eckert, 2012; Li et al., 2018). In this study, we use conditional mouse genetics coupled with histological and gene expression analyses to establish a novel role for PRMT5 during terminal hypertrophic chondrocyte differentiation in the vertebral growth plate and endochondral ossification in the spine. Mice harboring conditional deletion of *Prmt5* in osteochondral progenitor cells display scoliosis and perinatal lethality reminiscent of the natural history of infantile IS. Using inducible, conditional genetics in mice, we also demonstrate a continued role for PRMT5 function in vertebral growth plate and the cartilaginous endplate in adult mice. Overall, our work establishes PRMT5 as a critical factor for cartilaginous tissue development, endochondral ossification and homeostasis of the spine.

## RESULTS

### Conditional loss of *Prmt5* in the spine models infantile IS in mouse

**Fig. 1.**
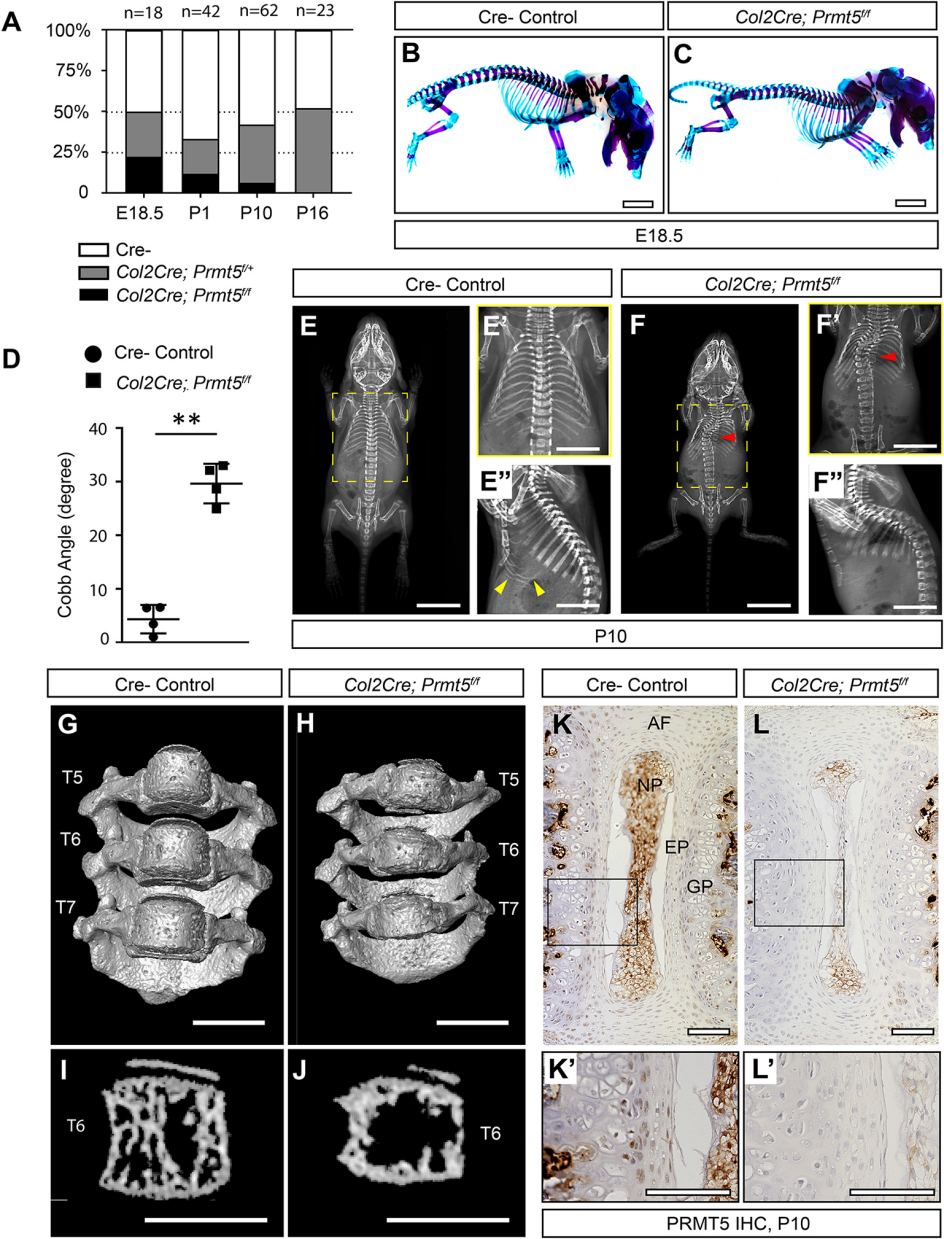
**Loss of *Prmt5* in osteochondral progenitor cells induces early-onset scoliosis in mice.** (A) *Col2Cre;Prmt5^f/f^* mutant mice were produced in Mendelian ratios at E18.5 (22.2%, *n*=18), but displayed progressive mortality prior to weaning (11.9%, *n*=42 at P1; 5.4%, *n*=62 at P10; 0%, *n*=23 at P16). (B,C) Skeletal preparations showed comparable size and normal patterning of the spine in *Col2Cre;Prmt5^f/f^* mutant mice at E18.5 compared with the littermate Cre^–^ controls (*n*=4 for each group). (D-F″) X-ray imaging analysis and quantification at P10 demonstrating early-onset thoracic scoliosis (F,F′, red arrowheads) and increased Cobb angle in mutant mice (D) (*n*=4 for each group; each dot represents one mouse analyzed, plotting with mean± s.d.; **P<0.01, two-tailed Student’s *t*-test). Sagittal X-ray image showing reduced ossification of the distal ribs in the mutant (F″) compared with a littermate control (E″, yellow arrowheads). (G-J) MicroCT scanning of the thoracic region of the spine (T5-T7) shows smaller vertebrae and a mildly wedged vertebral body at the curvature (T6) in mutant mice (H) compared with the controls (G) at P10. Sagittal sections of the microCT three-dimensional reconstruction of the thoracic vertebral bodies shows reduced bone formation within the vertebral body in the mutant mice (J) compared with controls (I) at P10. (K-L′) Immunohistochemical (IHC) analysis of PRMT5 in thoracic spine sections of control (K) and mutant (L) mice at P10. The boxes outline the areas shown at higher magnification in K′ and L′. Scale bars: 2 mm (B,C); 10 mm (E,F); 50 mm (E′,E″,F′,F″); 1 mm (G-J); 100 µm (K-L′). AF, annulus fibrosus; EP, endplate; GP, growth plate; NP, nucleus pulposus.

In order to test the role of PRMT5 in the spine, we crossed mice harboring a *Prmt5^f/f^* conditional allele (Norrie et al., 2016) to *Col2Cre* mice (Long et al., 2001). This *Col2Cre* allele demonstrates robust Cre activity in osteochondral progenitors and effectively labels most cartilaginous lineages of the spine (Zheng et al., 2019). At postnatal day (P)1, Rosa26-LacZ recombination can be observed in the IVD tissues, including the nucleus pulposus, endplate and annulus fibrosus, as well as the vertebral growth plate, periosteum and some newly formed trabecular bone tissues in the vertebra (Fig. S1). *Col2Cre;Prmt5^f/f^* mutant pups were produced at Mendelian ratios when assessed at embryonic day (E)18.5 (22.2%, *n=*18). However, mutant animals died of unknown causes prior to weaning (11.9% survival assessed at P1, *n=*42; 5.4% survival assessed at P10, *n=*62) (Fig. 1A). We did not recover any *Col2Cre;Prmt5^f/f^* mutants at P16 (0%, *n*=23). Two of the mutant mice that survived to P10 (*n*=4) were much smaller than the control mice (Fig. 1F), whereas the other two were comparable to the littermate controls, suggesting variable penetrance. At E18.5, whole-mount skeletal preparation revealed no obvious defects in the size, patterning and maturation of the skeleton in the *Col2Cre;Prmt5^f/f^* mutants compared with littermate controls (Fig. 1C; *n=*4 for each group). However, at P10, we observed IS-like thoracic scoliosis (Fig. 1F, red arrowhead) with an average Cobb angle of 30±4° in the *Col2Cre;Prmt5^f/f^* mutants (Fig. 1D; *n*=4/4). Lateral X-rays showed reduced signal attenuation in the distal ribs, indicative of a reduction in mineralized bone in mutant mice (Fig. 1F″) relative to that in aged-matched littermate controls (Fig. 1E″, yellow arrowheads). To gain more insight into the nature of the spine curvature, we performed microcomputed tomography (microCT) analysis of the thoracic region of the spine (T2-T12) of both *Col2Cre;Prmt5^f/f^* mutant and littermate control mice at P10. The rendered microCT dataset clearly illustrates the thoracic curvature observed in the mutant mice (Fig. 1G,H; Fig. S2, Movies 1 and 2). We also observed overall smaller vertebrae and some mild wedging of the vertebra at the apex of the curvature (Fig. 1H, T6; Fig. S2B, red arrow). Sagittal slices of the microCT data at the level of the T6 vertebrae showed a severe reduction in trabecular bone formation in the *Col2Cre;Prmt5^f/f^* mutant mice compared with the controls (Fig. 1I,J). Immunohistochemistry (IHC) against PRMT5 revealed robust protein expression in the vertebral growth plate and within the endplate and annulus fibrosus tissues of the IVD in wild-type mice at P10 (Fig. 1K-L′) and P1 (Fig. S3), which was consistently reduced in *Col2Cre;Prmt5^f/f^* mutant mice at these stages. We did not observe PRMT5 expression in the trabecular bone or cortical bone (Fig. S3C,C′), suggesting that PRMT5 has a limited role in committed osteoblast lineages.

### Loss of *Prmt5* in osteochondral progenitors results in asymmetric defects of endochondral ossification in the spine

**Fig. 2.**
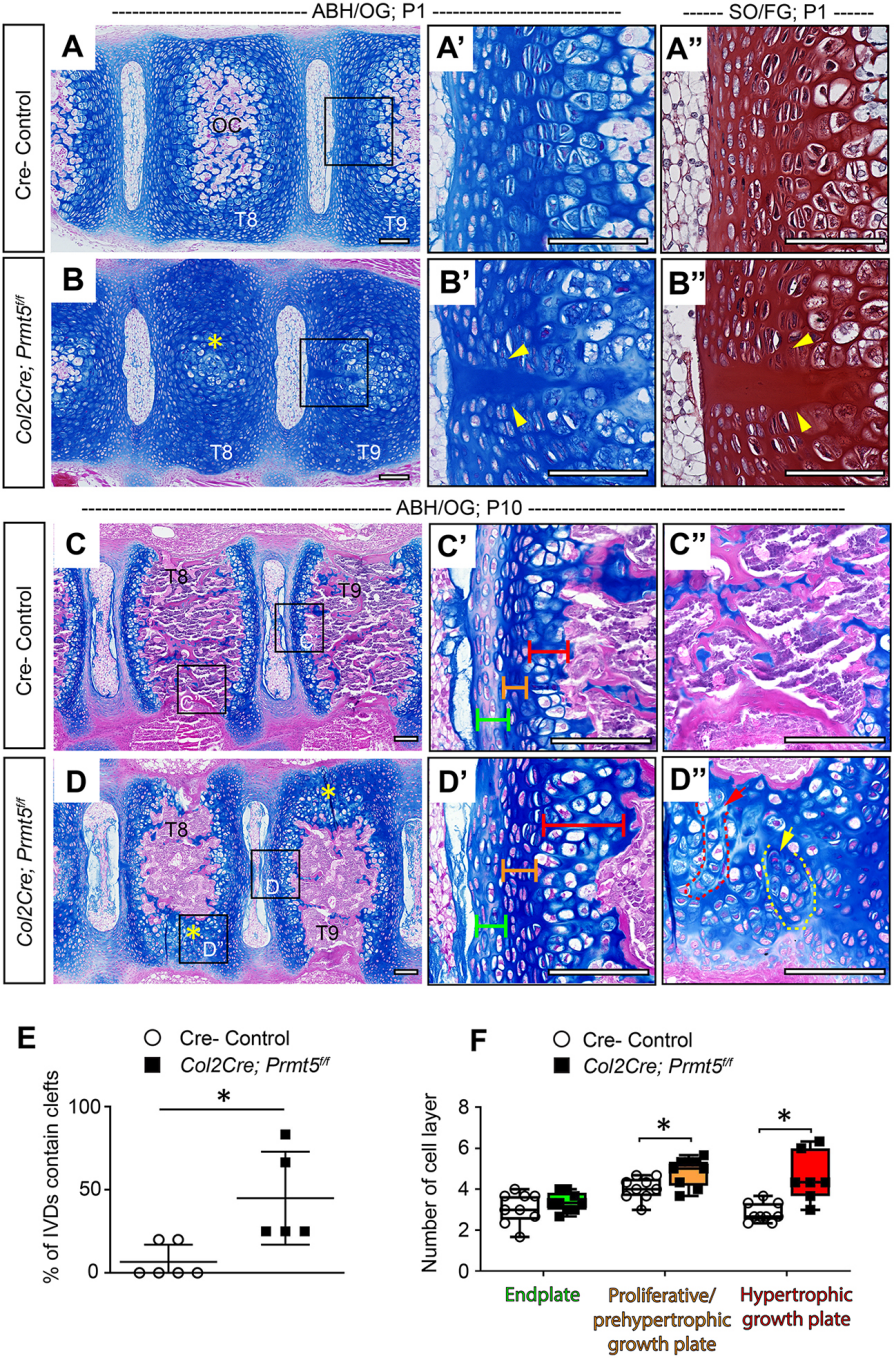
**Loss of *Prmt5* in osteochondral progenitor lineages of the spine results in defective ossification of the vertebral body.** (A-D) Alcian Blue Hematoxylin/Orange G (ABH/OG) staining of thoracic spines of Cre^–^ control (A,C) or *Col2Cre;Prmt5^f/f^* mutant (B,D) mice at P1 (A,B) and P10 (C,D). The boxes outline the areas shown at higher magnification in A′, A″, B′, B″, C′, C″, D′ and D″. (A″,B″) P1 adjacent sections stained with Safranin O/Fast Green (SO/FG). (B) Impaired ossification in *Col2Cre;Prmt5^f/f^* mutant vertebrae (yellow asterisk). (B′,B″) Representative images of midline clefts consistently observed in mutant endplate, but less common in Cre^–^ control mice (A′,A″) (yellow arrowheads; *n*=6 for controls and *n*=5 for mutants). (D) At P10, regions of asymmetric defects of bone formation observed in the vertebrae of *Col2Cre;Prmt5^f/f^* mutant mice (D, yellow asterisks), which are ossified in Cre^–^ control mice (C). The organization of chondrocytes in regions of persistent cartilage were found in columns (D″, red arrow/dashed outline) and clusters (D″, yellow arrow/dashed outline) (*n*=3 for each group). Endplate, proliferative/prehypertrophic growth plate and hypertrophic growth plate are labeled with green, orange and red brackets, respectively. The regions of the thoracic spine checked (T8, T9) are labeled on the vertebrae. (E) Quantification of the percentile of IVDs contain midline clefts in control and mutant IVD at P1 as shown in B-B″. Each dot represents one mouse analyzed, plotting with mean±s.d. (*n*=6 mice for controls and *n*=5 mice for mutants). Four to six thoracic IVDs were checked for each mouse. (F) Quantification of the number of cell layers of the endplate and growth plate in control and mutant mice at P10 as shown in C′ and D′. Each dot in the box and whisker plot represents the counts from a single IVD and adjacent growth plate analyzed. Each IVD and growth plate was analyzed three times and averaged, and at least two IVDs and growth plates were analyzed per mouse (*n*=3 mice for each group). *P<0.05, two-tailed Student's *t*-test. Scale bars: 100 µm.

Consistent with our observations by microCT (Fig. 1I,J), we observed impaired endochondral ossification of the thoracic vertebral bodies in *Col2Cre;Prmt5^f/f^* mutant mice at both P1 (Fig. 2B) and P10 (Fig. 2D) by histology. At P1, these mutant mice displayed a delay in the formation of the endochondral ossification center in the vertebral bodies, coupled with an increase in midline clefts in the IVD endplate and the vertebral growth plate (Fig. 2B′,B″, quantified in E). Interestingly, increased midline clefts were observed in conditional *Adgrg6/Gpr126* scoliosis mutant mice (Karner et al., 2015). These acellular midline clefts are likely indicative of delayed midline fusion of the cartilaginous anlagen during the withdrawal of the embryonic notochord from the regions of the developing vertebral body into the nucleus pulposus (Smith et al., 2011). At P10, we observed impaired endochondral ossification and reduced trabecular bone formation in the thoracic vertebral bodies of the mutant mice (Fig. 2D,D″). In some instances, mutant mice exhibited stark asymmetries of endochondral ossification even in adjacent vertebrae (Fig. 2D, yellow asterisks). We also observed a general disorganization of the chondrocytes, including the formation of columns or clusters within these areas of persistent cartilage in the vertebrae (Fig. 2D″, red and yellow dashed outlines).

We also observed consistent morphological alterations in some of the IVD tissues and the vertebral growth plate of the mutant mice at P10. In control mice, the IVD endplate contains several layers of flat cells that lightly stain with Alcian Blue (Fig. 2C′, green bracket). The proliferative/prehypertrophic zone of the control growth plate is made up of several layers of flat chondrocytes that organize into columns (Fig. 2C′, orange bracket), while the hypertrophic zone of the growth plate is composed of two to four layers of enlarged hypertrophic chondrocytes (Fig. 2C′, red bracket). In contrast, *Prmt5* mutant chondrocytes in the endplate appear larger, and the surrounding matrix is more intensely stained by Alcian Blue (Fig. 2D′, green bracket). Quantitative analysis of these cells revealed an increased number of cell layers in the hypertrophic zone of the vertebral growth plate, but not in the IVD endplate of the *Col2Cre;Prmt5^f/f^* mutant mice (Fig. 2F). We also quantified a minor increase in the number of cell layers in proliferative/prehypertrophic zone of the growth plate (Fig. 2F). We consistently observed that the inner and outer layers of the annulus fibrosus in *Col2Cre;Prmt5^f/f^* mutant mice stained more intensely with Alcian Blue than those in the littermate controls (Fig. S4A,B). To determine whether these morphological alternations were region specific, we repeated the same analyses in the lumbar spine, observing identical phenotypes in both regions of the spine (Fig. S5B,B′). Collectively, these data suggest a model in which PRMT5 is critical for several aspects of vertebral body development, including chondrocyte differentiation and endochondral ossification along the entire spine axis.

### Loss of *Prmt5* in osteochondral progenitors results in altered gene expression of cartilage components and accumulation of COLX in the IVD and growth plate

**Fig. 3.**
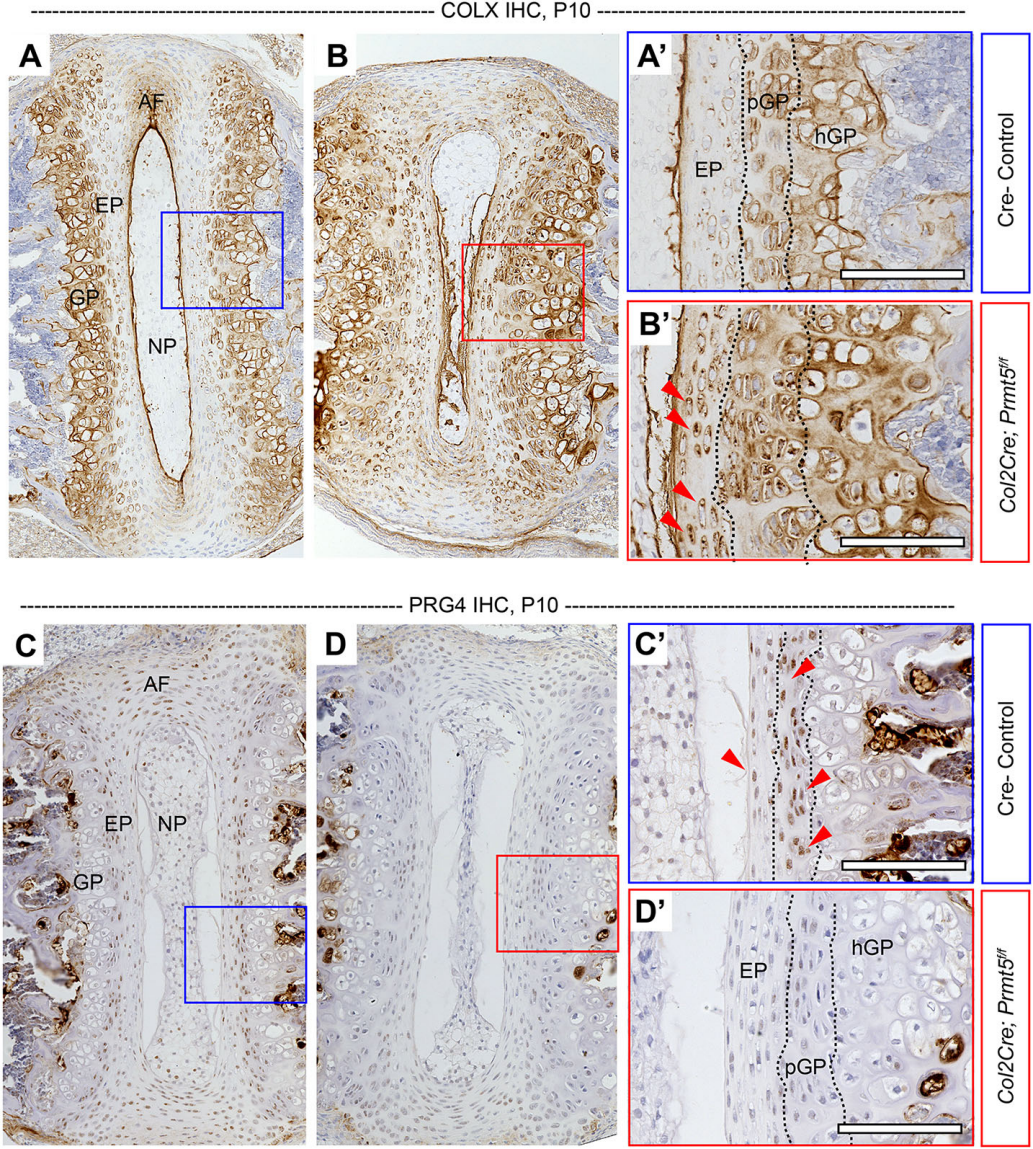
**Loss of *Prmt5* in osteochondral progenitors results in induced expression of COLX and depleted expression of PRG4 in the intervertebral disc.** (A-D′) IHC analysis of type X collagen (COLX) (A,B) and proteoglycan 4 (PRG4) (C,D) in thoracic spine sections of Cre^–^ control (A,C) or *Col2Cre;Prmt5^f/f^* mutant (B,D) mice at P10. The boxes outline the areas shown at higher magnification in A′-D′. Mutant mice demonstrated increased COLX signal in the endplate (B′, red arrowheads) and growth plate, and diminished PRG4 signal in the endplate and growth plate (D′), which was consistently observed in the Cre^–^ control mice (C′, red arrowheads) (*n*=3 mice for each group). Scale bars: 100 µm. AF, annulus fibrosus; EP, endplate; GP, growth plate; hGP, hypertrophic growth plate; NP, nucleus pulposus; pGP, proliferative growth plate.

We next assayed several established regulators of chondrocyte differentiation (Long and Ornitz, 2013) in order to assess potential mechanisms for PRMT5-dependent regulation of spine development. IHC analysis of the hypertrophic chondrocyte marker COLX revealed ectopic, increased expression in both the proliferative and hypertrophic zones of the growth plate in *Col2Cre;Prmt5^f/f^* mutant mice (Fig. 3B,B′) in comparison to that in the control mice (Fig. 3A,A′). Increased COLX expression is also observed in the endplate (Fig. 3B,B′), but not in the annulus fibrosus (Fig. S4D) of the mutant mice. However, the expression of proteoglycan 4 (PRG4/Lubricin), a common marker of healthy cartilage and IVD (Jay, 1992; Jay and Waller, 2014; Teeple et al., 2015), was remarkably absent in the vertebral growth plate, the IVD endplate and the annulus fibrosus of *Col2Cre;Prmt5^f/f^* mutant mice at P10 (Fig. 3D,D′; Fig. S4F). The expression of SOX9, a key transcriptional regulator of chondrogenesis, was also reduced in the endplate of mutant mice (Fig. S6C-D′), but was not obviously affected in the proliferative growth plate. Hypertrophic chondrocytes of the vertebral growth plate downregulate *Sox9* expression during the process of terminal hypertrophic chondrocyte differentiation (Fig. S6C′); however, *Col2Cre;Prmt5^f/f^* mutant mice continued to express SOX9 in some of these phenotypically hypertrophic cells of the growth plate (Fig. S6D′). The annulus fibrosus typically expressed low levels of SOX9 (Fig. S4I); however, this expression was greatly diminished in mutant mice (Fig. S4J). Interestingly, the loss of SOX9 expression did not strongly correlate with reduced type II collagen (COLII) expression, which is an established direct target of SOX9 (Bell et al., 1997), in *Col2Cre;Prmt5^f/f^* mutant mice at P10 (Fig. S4G,H; Fig. S6A-B′). Together, our results suggest that PRMT5 regulates the differentiation of the cartilaginous tissues of the IVD and growth plate in the spine.

### PRMT5 regulates the expression of critical factors of chondrocyte differentiation during endochondral ossification in the spine

**Fig. 4.**
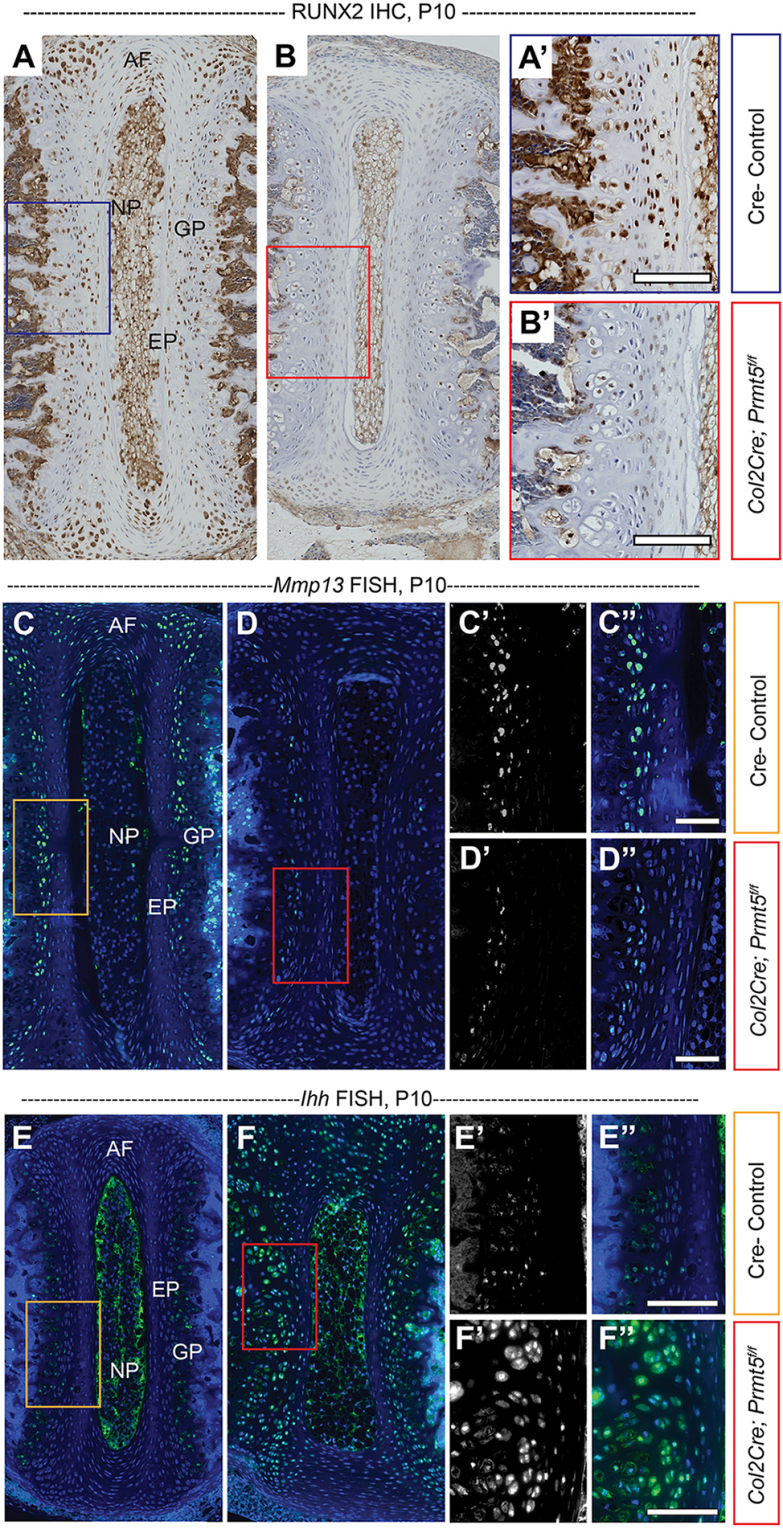
**PRMT5 regulates the expression of RUNX2, *Mmp13* and *Ihh*.** (A,B) IHC analysis of RUNX2 in thoracic spine sections of Cre^–^ control (A) or *Col2Cre;Prmt5^f/f^* mutant (B) mice at P10, demonstrating reduced RUNX2 expression in the endplate and growth plate of the mutant mice. The boxes outline the areas shown at higher magnification in A′ and B′. (C,D) Fluorescent *in situ* hybridization (FISH) analysis of *Mmp13* in thoracic spine sections of Cre^–^ control (C) or *Col2Cre;Prmt5^f/f^* mutant (D) mice at P10, with 4′,6-diamidino-2-phenylindole (DAPI; nuclei) in blue. Reduced *Mmp13* signal was detected in the growth plate of the mutant mice. C′ and D′ are grayscale *Mmp13* fluorescent *in situ* channels; C, C″, D and D″ are pseudocolored merged channels. The boxes outline the areas shown at higher magnification in C′, C″, D′ and D″. (E,F) FISH analysis of *Ihh* in thoracic spine sections of Cre^–^ control (E) or *Col2Cre;Prmt5^f/f^* mutant (F) mice at P10. Induced expression of *Ihh* was detected in the growth plate of the mutant mice. E′ and F′ are grayscale *Ihh* fluorescent *in situ* channels; E, E″, F and F″ are pseudocolored merged channels. The boxes outline the areas shown at higher magnification in E′, E″, F′ and F″ (*n*=3 for each group). Scale bars: 100 µm. AF, annulus fibrosus; EP, endplate; GP, growth plate; NP, nucleus purposes.

We next set out to determine the mechanisms by which PRMT5 regulates hypertrophic chondrocyte differentiation and endochondral bone formation. RUNX2 is a critical driver of terminal differentiation of hypertrophic chondrocytes and osteoblast differentiation in mouse (Long and Ornitz, 2013; Takarada et al., 2013), and acts in part through the activation of *Mmp13* (Inada et al., 1999; Komori, 2010a, 2018). Using IHC, we showed that RUNX2 is typically expressed in some cells of the IVD endplate and the inner layer of the annulus fibrosus (Fig. 4A,A′; Fig. S4K). RUNX2 is also expressed in some cells of the proliferative growth plate and in the majority of hypertrophic growth plate cells, as well as some cells of the trabecular bone, by the age of P10 (Fig. 4A,A′). In contrast, the expression of RUNX2 was markedly diminished in all these tissues in *Col2Cre;Prmt5^f/f^* mutant mice at P10 (Fig. 4B,B′; Fig. S4L). Interestingly, we did not observe an obvious loss of RUNX2 expression earlier in development in *Col2Cre;Prmt5^f/f^* mutant mice at P1 (Fig. S7A-B′). Next, we performed fluorescent *in situ* hybridization (FISH) using an *Mmp13*-specific riboprobe on thoracic spine sections. At P10, there is severe reduction in *Mmp13* expression in the vertebral growth plate and trabecular bone in *Col2Cre;Prmt5^f/f^* mutant mice (Fig. 4D-D″), compared with robust expression in the hypertrophic growth plate in control mice (Fig. 4C-C″). We observed sparse expression of *Mmp13* in the annulus fibrosus in control mice, which is diminished in mutant mice (Fig. S4M,N). The loss of *Mmp13* expression is also observed in the presumptive ossification center of the vertebrae at P1 in *Col2Cre;Prmt5^f/f^* mutant mice (Fig. S7D-D″). Conversely, at P1, we observed ectopic *Mmp13* signal within the nucleus pulposus and the endplate in mutant mice, which was not observed in control mice (Fig. S7C-C″). The consistent reduction of RUNX2 and its effector, *Mmp13*, in the vertebral growth plate of *Col2Cre;Prmt5^f/f^* mutant mice demonstrates a mechanistic role for PRMT5 in the regulation of terminal hypertrophic chondrocyte differentiation and endochondral ossification during perinatal development of the spine.

To better understand the regulatory mechanisms of PRMT5 during endochondral bone formation, we next assayed *Ihh* expression. *Ihh* signaling is crucial to maintain chondrocyte proliferation and, at the same time, negatively regulate hypertrophic chondrocyte differentiation by regulation of PTHrP expression (Long and Ornitz, 2013). However, *Ihh* is also required to promote chondrocyte hypertrophy by induction of COLX expression through RUNX2/Smad protein-dependent processes (Amano et al., 2014; Mak et al., 2008). At P1, FISH using an *Ihh*-specific riboprobe shows comparable expression pattern in control and *Col2Cre;Prmt5^f/f^* mutant mice, suggesting that PRMT5 is not required for early stages of hypertrophic chondrocyte differentiation (Fig. S7E,F), which is further supported by typical RUNX2 expression in mutant mice at this stage (Fig. S7A-B′). However, at P10, we observed a dramatic increase in ectopic *Ihh* expression in the mutant mice, including ectopic expression of *Ihh* in the proliferative and hypertrophic growth plate, as well as in some cells of the endplate and annulus fibrosus regions of the IVD (Fig. 4F-F″; Fig. S4P), compared with only sparse expression in the prehypertrophic/hypertrophic chondrocytes in control mice (Fig. 4E-E″). Together, our results demonstrate that PRMT5 regulates multiple signaling inputs of terminal hypertrophic differentiation and endochondral ossification, specifically via positive regulation of RUNX2 and *Mmp13* expression and through negative regulation of *Ihh* expression during perinatal development.

### PRMT5 regulates *Bmp4* expression in the spine

Loss of *Prmt5* in early limb bud mesoderm and lung tissue induces ectopic, elevated *Bmp4* expression, which is accompanied by increased apoptosis in these tissues (Li et al., 2018; Norrie et al., 2016). Consistent with these observations, we also observed increased ectopic *Bmp4* signal in the inner layer of the annulus fibrosus and the growth plate in the *Col2Cre;Prmt5^f/f^* mutant mice at P10, which was not present in controls (Figs S4Q,R and S8A-B′). We did not observe upregulation of *Bmp4* expression in the growth plate at P1 in either genotype (Fig. S8C-D′), suggesting that this increased expression accumulates during perinatal development. We also detected a mild increase in cell death in the annulus fibrosus, endplate and growth plate regions of the *Col2Cre;Prmt5^f/f^* mutant mice at both P1 and P10 (Fig. S9). Taken together, these data demonstrate that, as in other tissues and organs, PRMT5 is required for the inhibition of *Bmp4* expression in the cartilaginous tissues of the spine.

### PRMT5 is not required in the mature osteoblast lineages for spine development and stability

Given the clear alterations in endochondral bone formation in *Col2Cre;Prmt5^f/f^* mutant mice, we next sought to determine whether PRMT5 also functions in committed bone-forming lineages. To address this, we utilized an *OcCre* (*BglapCre*) transgenic mouse to specifically remove PRMT5 function in mature osteoblasts (Zhang et al., 2002). The conditional mutant mice were born at Mendelian ratios and remain adult viable, displaying no obvious phenotypes (*n*=7). X-ray analysis showed no scoliosis in the *OcCre;Prmt5^f/f^* mutant mice at P10 (Fig. S10A,B, *n*=4) or at 2 months of age (*n*=3), and histological analysis at P10 showed no obvious alterations of spine tissues (Fig. S10C,D). Furthermore, we did not detect PRMT5 expression in the cortical or trabecular bone of the spine (Fig. S3C,C′). Taken together, we conclude that PRMT5 functions exclusively in chondrocyte lineages to promote endochondral ossification and does not have an obvious role in mature osteoblast lineages.

### PRMT5 has a role in homeostasis of the cartilaginous tissues of the adult spine

Chondrocytes regulate spine homeostasis by maintaining a dynamic balance between synthesis and degradation of extracellular matrix components in the cartilaginous tissues of the spine. To gain insight into whether PRMT5 may have a continuous role in homeostasis of the adult spine, we next assayed the expression of PRMT5 in wild-type thoracic spine sections at 6 weeks and 4 months of age. We observed low-level PRMT5 expression in a group of cells bordering the junction between the IVD and the vertebral growth plate at both time points (Fig. S11). Close examination of this region of the IVD revealed that PRMT5-positive cells reside in various tissues, including the outer layer of the annulus fibrosus, the border of the endplate and within the growth plate (Fig. S11A′,B′,C′).

**Fig. 5.**
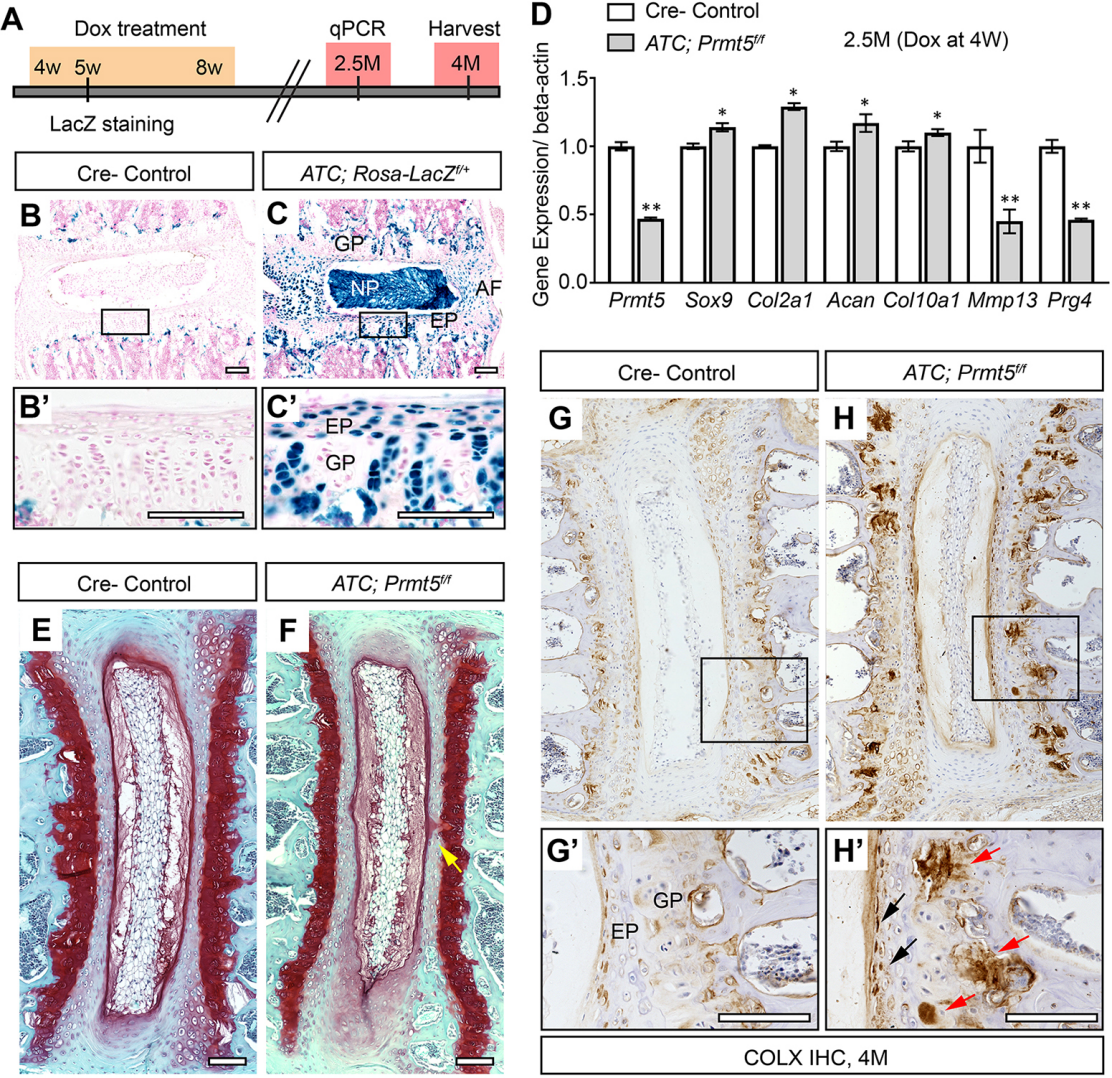
**Postnatal ablation of *Prmt5* in cartilaginous tissues of the spine resulted in alterations** **in** **normal gene expression.** (A) Schematic of the protocol for postnatal recombination of the floxed *Prmt5* allele used for these experiments. (B,C) Beta-galactosidase staining in thoracic spine sections of Cre^–^ control (B) or *ATC;Rosa-LacZ*^*f/+*^ (C) mice at 5 weeks of age shows that effective recombination is achieved within the IVD and growth plate at 1 week post-Dox induction. The boxes outline the areas shown at higher magnification in B′ and C′. (D) Real-time RT-PCR analysis of IVD and growth plate tissues harvested at 2.5 months of age demonstrates effective reduction in *Prmt5* as well as *Mmp13* and *Prg4*. Bars represent mean±s.d. (*n*=3 for each group). *P<0.05, **P<0.01, two-tailed Student's *t*-test. (E,F) Safranin O/Fast Green staining in thoracic spine sections of Cre^–^ control (E) or *ATC;Prmt5^f/f^* mutant (F) mice harvested at 4 months of age after Dox treatment. No obvious histopathology was observed except for the occurrence of minor clefts of the endplate and growth plate in the mutant mice (F, yellow arrow). (G,H) IHC analysis of COLX protein in thoracic spine sections from Cre^–^ control (G) or *ATC;Prmt5^f/f^* mutant (H) mice at 4 months of age. The boxes outline the areas shown at higher magnification in G′ and H′. Increased and ectopic COLX signal was observed in the endplate (H′, black arrows) and growth plate (H′, red arrows) of the mutant mice (*n*=3 for each group). Scale bars: 100 µm. AF, annulus fibrosus; EP, endplate; GP, growth plate; NP, nucleus pulposus.

Because PRMT5 continues to be expressed in adult mice, we tested whether PRMT5 is required for the homeostasis of the spine. To specifically remove PRMT5 function in cartilaginous tissues of the spine after weaning, we crossed *Prmt5^f/f^* mice with an *Acan* enhancer-driven, tetracycline-inducible Cre (*ATC*) transgenic mouse, which targets committed chondrocyte lineages (Dy et al., 2012), and induced recombination from 4 to 8 weeks of age (Fig. 5A). Beta-galactosidase staining in *ATC;Rosa-LacZ^f/+^* mice revealed near complete recombination in nucleus pulposus, endplate and annulus fibrosus of the IVD, as well as over 50% recombination in vertebral growth plate at 1 week postinjection (5 weeks of age) (Fig. 5C). At 6 weeks postinduction (2.5 months of age), we isolated IVDs and adjacent growth plates from the thoracic and lumbar spines of both control and mutant mice, and generated cDNA libraries from these tissues. Real-time reverse transcription PCR (RT-PCR) analysis revealed ∼2-fold reduction in *Prmt5* expression, as well as ∼2-fold reduction in the expression of *Mmp13* and *Prg4* (Fig. 5D), respectively, in the mutant mice, which is consistent with our analysis of these genes in the IVD and growth plate when *Prmt5* is ablated during embryonic development in *Col2Cre;Prmt5^f/f^* mutant mice. In contrast to our observations in *Col2Cre;Prmt5^f/f^* mutant mice, the expression of *Sox9*, *Col2a1*, *Acan* and *Col10a1* was not strongly affected in *ATC;Prmt5^f/f^* mutant mice with this induction strategy (Fig. 5D). Moreover, at 4 months of age (3 months postinduction), there were no overt signs of degenerative histopathology in *ATC;Prmt5^f/f^* mutant IVD (Fig. 5F), with the exception of a minor increase in acellular clefts at the midline of the endplate (Fig. 5F, yellow arrow), which we occasionally observe in wild-type IVD as well (*P*=0.047; two-tailed Student’s *t-*test; *n*=3 mice for each group). However, we consistently observed increased, ectopic COLX expression in the IVD endplate and vertebral growth plate in *ATC;Prmt5^f/f^* mutant mice (Fig. 5H,H′). We did not observe scoliosis in *ATC;Prmt5^f/f^* mutant mice when induced from either 2 or 4 weeks of age by dorsal X-ray imaging (Fig. S12). Taken together, our data show that loss of PRMT5 in cartilaginous tissues of the adult spine results in reduced expression of normal extracellular matrix component *Prg4*, coupled with increased expression of the hypertrophic chondrocyte marker COLX and decreased expression of *Mmp13*. This suggests that PRMT5 has a continuous role in the regulation of hypertrophic chondrocyte turnover in the growth plate in adult mice. Our study demonstrates a novel role for PRMT5 in maintaining homeostasis of cartilaginous tissues in adult spine and defines a limited temporal window during perinatal development for the regulation of spine stability.

## DISCUSSION

The formation of bony tissues of vertebral bodies, like the long bones, occurs via a process of endochondral ossification, by which hypertrophic chondrocytes are replaced by, and in some cases contribute to, bone-forming osteoblast lineages (Aghajanian and Mohan, 2018). Our findings indicate that PRMT5 has a crucial role in the process of terminal hypertrophic chondrocyte differentiation and subsequently in endochondral bone formation. We demonstrate functions of PRMT5 in positive regulation of *Mmp13* and RUNX2 expression and in negative regulation of *Ihh* expression. We also demonstrate that loss of PRMT5 in osteochondral progenitors results in the expansion of hypertrophic chondrocytes in the vertebral growth plate; however, ossification of the vertebrae is severely impaired during perinatal development, mimicking an *Mmp13* loss-of-function phenotype (Inada et al., 2004). Finally, we show that PRMT5 regulation of perinatal endochondral bone formation is a potential mechanism underlying infantile IS. Taken together, these results establish PRMT5 as a fundamental regulator of terminal hypertrophic chondrocyte differentiation and endochondral bone formation, as well as a critical regulator of perinatal spine integrity.

### Regulation of terminal hypertrophic chondrocyte differentiation and endochondral bone formation by PRMT5

**Fig. 6.**
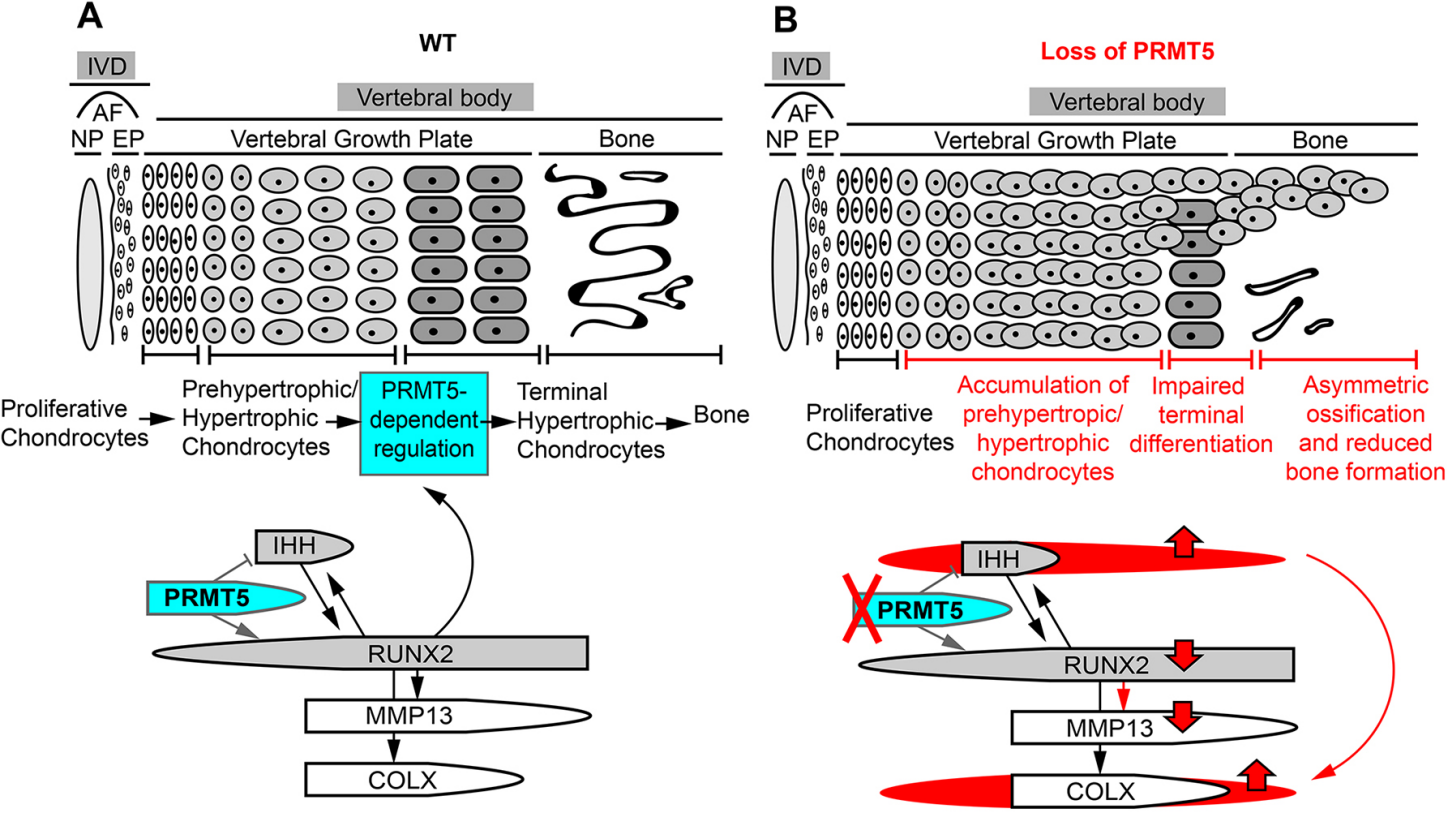
**A model of PRMT5 in regulating terminal differentiation of hypertrophic chondrocytes in the perinatal vertebral growth plate of the spine.** (A) PRMT5 expressed in proliferative and prehypertrophic chondrocytes functions to regulate chondrocyte hypertrophic differentiation by controlling RUNX2 and IHH signaling. PRMT5 is required for maintaining postnatal expression of RUNX2, which is a key regulator of terminal differentiation of hypertrophic chondrocytes, and a positive regulator of MMP13 expression. PRMT5 also negatively regulates the expression of *Ihh*. (B) *Prmt5* deletion in cartilaginous tissues of the spine leads to expanded *Ihh* expression in the perinatal growth plate and promotes the expression/accumulation of COLX. It also results in reduced RUNX2 and *Mmp13* expression, which inhibits the terminal differentiation of the hypertrophic chondrocytes. These two processes synergistically lead to persistent hypertrophic growth plate and reduced bone formation in perinatal mouse vertebrae. AF, annulus fibrosus; EP, endplate; IVD, intervertebral disc; NP, nucleus pulposus; WT, wild type.

RUNX2 is one of the most important regulators of hypertrophic chondrocyte differentiation and endochondral bone formation (Komori, 2010b, 2018). *Runx2*-deficient mice exhibit a complete loss of mature osteoblasts and endochondral bone formation (Chen et al., 2014; Ducy et al., 1997; Takarada et al., 2013). RUNX2 has been shown to directly regulate *Col10a1*, *Mmp13* and *Vegfa* expression (Hess et al., 2001; Li et al., 2011; Takahashi et al., 2017; Zelzer et al., 2001), and therefore plays a crucial role in driving chondrocyte differentiation and endochondral ossification (Fig. 6A). *Mmp13*, a direct target of RUNX2 (Fig. 6A), is also required for these processes, as mice lacking *Mmp13* display expanded hypertrophic zone and a delay in endochondral ossification in the long bone (Inada et al., 2004; Stickens et al., 2004). Here, we found that *Col2Cre;Prmt5^f/f^* mutant mice displayed an obvious reduction in RUNX2 and *Mmp13* expression in proliferative and hypertrophic chondrocytes of the perinatal growth plate. At the same time, *Col2Cre;Prmt5^f/f^* mutant mice showed a general expansion of the growth plate, as marked by COLX expression. These results suggest that PRMT5 is not critical for the commencement of hypertrophic differentiation. Instead, it appears to be crucial for terminal hypertrophic chondrocyte differentiation, specifically for processes important for the removal of COLX-positive hypertrophic cells, which is well established as a phenotype impaired in *Runx2* and *Mmp13* mutant mice. We have not addressed how hypertrophic chondrocyte differentiation is able to proceed without these key regulators of terminal chondrocyte differentiation. It is possible that undetectable levels of *RUNX2* and *Mmp13* are sufficient to promote some endochondral ossification, albeit in a severely delayed manner, or that other RUNX family members or co-factors, such as RUNX3, MEF2C or FOXA2, are compensating (Arnold et al., 2007; Tan et al., 2018; Yoshida et al., 2004).

IHH is another important regulator of hypertrophic chondrocyte differentiation. It can directly promote chondrocyte proliferation and inhibit premature hypertrophy of these proliferative chondrocyte via an IHH/PTHrP negative-feedback loop (Long and Ornitz, 2013). It can also promote hypertrophy and induce COLX expression in a PTHrP-independent manner (Amano et al., 2014; Mak et al., 2008). We observed that *Col2Cre;Prmt5^f/f^* mutant mice displayed ectopic expansion of *Ihh* expression throughout the hypertrophic growth plate, proliferative growth plate and some cells within the endplate of IVD at perinatal stage (P10) but not neonatal stage (P1). Interestingly, these expanded regions of *Ihh* expression overlap with the regions of ectopic, expanded COLX expression. These results again suggest that PRMT5 is dispensable for chondrocyte proliferation and the early maturation of hypertrophic chondrocyte differentiation; however, it is required to restrict *Ihh* expression in the IVD and growth plate during perinatal development. Increased *Ihh* expression may contribute to the increased production and accumulation of COLX-positive cells in the growth plate. We speculate that the misregulation of *Mmp13* and *Ihh* in *Col2Cre;Prmt5^f/f^* mutant mouse spines act synergistically to promote the accumulation of hypertrophic chondrocytes in the growth plate by inhibition of processes driving terminal chondrocyte differentiation, which consequently leads to delays and reductions in endochondral bone formation during perinatal skeletogenesis. The mechanisms of PRMT5-dependent regulation of *Mmp13* and *Ihh* expression remain to be determined.

In a parallel study, we found that PRMT5 is also required for hypertrophic chondrocyte differentiation in long bones, with striking similarities in the expression of some of these genes (Ramachandran et al., 2019). The proliferative growth plate of the long bone in the *Prmt5* mutant mice is disorganized and shows reduced expression of *Col2a1* and *Sox9*, while the hypertrophic chondrocytes also undergo a blockage in terminal differentiation, highlighted by reduced expression of RUNX2 and *Mmp13*, and increased expression of *Ihh* (Ramachandran et al., 2019). In addition, PRMT5 has been shown to regulate *Bmp4* expression in several contexts. For example, loss of *Prmt5* in mouse mesenchymal progenitor cells led to upregulated *Bmp4* in mouse limb (Norrie et al., 2016). In agreement, *Col2Cre;Prmt5^f/f^* mutant mice also displayed ectopic elevation of *Bmp4* expression in the hypertrophic growth plate. Interestingly, *in vitro* studies have shown that *Bmp4* can stimulate chondrocyte hypertrophy and induce COLX expression (Clark et al., 2009; Hatakeyama et al., 2004; Steinert et al., 2009). Several other studies show that overexpression of *Ihh* in mouse leads to upregulation of *Bmp4* expression in perichondrium (Minina et al., 2001), while *Bmp4* expression is dramatically decreased in embryonic bone tissues of a *Runx2* null mice (James et al., 2006). Collectively, these studies find that *Bmp4* acts as a positive regulator of hypertrophic chondrocyte differentiation. However, whether PRMT5-dependent regulation of *Bmp4* expression is a driver of defective terminal hypertrophic chondrocyte differentiation remains to be determined.

Interestingly, the formation of the cartilaginous templates and some ossification occurs normally in these conditional *Col2Cre;Prmt5^f/f^* mutant mice, despite *Prmt5* deletion in osteochondral progenitor lineages. This is in contrast to the more severe loss of cartilage template in *Prx1Cre;Prmt5^f/f^*, resulting in a dramatic reduction in the mouse forelimb (Norrie et al., 2016). Taken together, these findings support a model with distinct mechanistic roles for PRMT5 in the initiation of early chondrocyte progenitors and the terminal differentiation of hypertrophic chondrocytes.

### PRMT5 regulates homeostasis of the cartilaginous tissues in adult spine

The IVD, largely composed of cartilaginous or fibrocartilaginous tissues, is an important component of spinal physiology. IVD homeostasis is characterized by slow rates of protein synthesis and breakdown of extracellular matrix component, which is tightly regulated by anabolic (e.g. SOX9, TGF protein) and catabolic (e.g. MMPs, ADAMTS protein) factors (Dowdell et al., 2017). For instance, postnatal loss of *Sox9* leads to degenerative changes and global alterations of gene expression in the IVD (Henry et al., 2012). Meanwhile, the translation of mechanical loading signals activates TGFβ signaling, which promotes IVD homeostatic processes (Bian et al., 2017). Recent studies also show that *Runx2* is required for postnatal IVD maintenance (Alvarez-Garcia et al., 2018; Liao et al., 2019). Here, we found that postnatal loss of PRMT5 in cartilaginous lineages of the spine resulted in alterations in the normal gene expression in IVD and vertebral growth plate, including reduced expression of *Prg4* as well as increased expression of COLX, which are both markers of early-onset degenerative disc.

These findings demonstrate that PRMT5 has an important role in the homeostasis of cartilaginous tissues of the spine. It will be important to determine whether ablation of PRMT5 in cartilaginous lineages of mature adult mice can generate susceptibility to the onset of more severe histopathological changes in the IVD and growth plate due to trauma or aging in mice. Interestingly, a study using bromodeoxyuridine labeling demonstrated that some IVD or IVD-adjacent regions, such as the perichondrium and sites of annulus fibrosus ligament anchoring, display label-retaining cells indicative of an adult stem cell niche (Henriksson et al., 2009). Importantly, these regions closely overlap with expression of PRMT5 in the adult mouse spine, suggesting a role for PRMT5 in maintaining adult progenitor/stem cell pools important for the homeostasis of the IVD and growth plate in adult mice.

### Asymmetrical properties of the spine result in scoliosis

Functional studies informed by human genetic studies of IS using animal models show that defects in connective tissue and cartilaginous tissues of the spine underlie the pathogenesis of some forms of IS in humans (Karner et al., 2015; Kim et al., 2013). Asymmetries in vertebral body growth and in mechanical properties of the bony prominences, cartilaginous joints of the vertebral bodies and musculature attachments of the spinal column have long been hypothesized to underlie the formation of IS (Liu and Gray, 2018). Our demonstration of IS-like scoliosis during early perinatal development in *Col2Cre;Prmt5^f/f^* mutant mice phenotypically models infantile-onset IS in humans. This is phenotypically distinct from congenital scoliosis, which is characterized by failure of segmentation, formation or dysplasia in one or more vertebral units (Pahys and Guille, 2018), whereas IS patients do not display overt vertebral malformations (Cheng et al., 2015). In the *Col2Cre;Prmt5^f/f^* scoliosis model presented here, we did not observe spinal deformity during embryonic development (E18.5), but find a clear thoracic spinal curvature present at P10, coupled with loss of bone formation in the ribcage and perinatal lethality. The natural history of this mouse model partially recapitulates aspects of severe cases of infantile IS in humans. The *Col2Cre;Prmt5^f/f^* mutant mice do not display vertebral dysplasia prior to or at birth. However, we do observe mild wedging of the vertebral body at the apex of the thoracic curvature at P10. Given that bone deposition is highly responsive to mechanical forces, we suggest that the onset of scoliosis is caused by defects in endochondral ossification of the vertebral bodies, which generate anisotropic mechanical loading of the spine, which initiates the onset of scoliosis and vertebral wedging. This model is supported by our observation of a complete lack of scoliosis in conditional *ATC;Prmt5^f/f^* mutant mice recombined at 2 or 4 weeks of age, which also display alterations in terminal chondrocyte differentiation, yet exhibit a mature spinal column that has already undergone substantial ossification at the induction of recombination. In conclusion, we suggest a model wherein infantile IS may be induced by subtle defects and/or delays in endochondral ossification during perinatal development, and that severe cases resulting in respiratory stress may reflect delayed endochondral ossification of the floating ribs. Finally, this study further underscores the role of cartilaginous tissues and regulation of chondrocyte differentiation in the pathogenesis of IS, which is a common theme in other genetic mouse models displaying IS (Liu and Gray, 2018), including conditional mutant mice of the *Gpr126*/*Adgrg6* (Karner et al., 2015), *Sox9* (Henry et al., 2012) and *Gdf5/6* (Settle et al., 2003) genes. Our analysis of the role of PRMT5 in this process extends our knowledge of the mechanisms maintaining spine stability towards regulation of endochondral ossification. In light of these results, high-resolution, low-dose X-ray imaging and analysis of bone quality, coupled with targeted analysis of the *PRMT5* locus, in infantile IS patients is warranted.

## MATERIALS AND METHODS

### Mouse strains

Animal studies comply with all relevant institutional and national animal welfare laws, guidelines and policies for the care and use of experimental animals, and were approved by the Institutional Animal Care and Use Committee at The University of Texas at Austin (protocol AUP-2018-00276). All mouse strains, including *Prmt5^f/f^* [Prmt5^tm2c(EUCOMM)Wtsi^] (Norrie et al., 2016), *Col2Cre* (Long et al., 2001), *ATC* (Dy et al., 2012), *OcCre* (Zhang et al., 2002) and *Rosa-LacZ* (Soriano, 1999), were described previously. Doxycycline (Dox) was administered to *ATC;Prmt5^f/f^* and littermate controls or *ATC;Rosa-LacZ^f/+^* mice via intraperitoneal (IP) injections by two strategies: (1) starting at 2 weeks of age, once/week (10 mg Dox/kg body weight) for 4 continuous weeks; or (2) starting at 4 weeks of age, once/week (10 mg Dox/kg body weight) for 4 continuous weeks. Mice were harvested at P1, P10, 6 weeks, 2.5 months and 4 months of age for tissue analysis.

### Analyses of mice

Skeletal preparations were performed as previously described (Allen et al., 2011). Radiographs of mouse skeleton were generated using a Kubtec DIGIMUS X-ray system (Kubtec T0081B) with auto exposure under 25 kV. Cobb angle was measured on high-resolution X-ray images with ImageJ (https://imagej.nih.gov/ij/), as previously described (Cobb, 1948). Histological analysis was performed on thoracic (T5-T12) and lumbar (L1-L5) spines fixed in 10% neutral-buffered formalin for 3 days at room temperature, followed by 1-week decalcification in Formic Acid Bone Decalcifier (Immunocal, StatLab). After decalcification, bones were embedded in paraffin and sectioned at 5 μm thickness. Alcian Blue Hematoxylin/Orange G (ABH/OG) and Safranin O/Fast Green (SO/FG) staining was performed following standard protocols. Quantification of IVD clefts and cell layers was performed on ABH/OG-stained sections of six control and five mutant mice. Four to six thoracic IVDs were analyzed per mouse. MicroCT analysis was performed on thoracic regions of the spine (T2-T12) in control and mutant mice at P10. The spines were fixed in 10% neutral-buffered formalin for 3 days at room temperature, thoroughly washed, and scanned on an Xradia at 100 kV at 9.65 μm true voxel resolution at the University of Texas High-Resolution X-ray Computed Tomography Facility (http://www.ctlab.geo.utexas.edu/). IHC analyses were performed on paraffin sections of thoracic spine after antigen retrieval using 10 mM Tris-HCl and 1 mM EDTA (with 0.05% Triton X-100, pH 9.0, heated in a 75°C water bath for 5 min) (PRMT5 and RUNX2), 4 mg/ml pepsin (COLII and COLX), 100 μg/ml hyaluronidase (PRG4) or 10 μg/ml proteinase K (SOX9). Colorimetric development was performed with the following primary antibodies: anti-PRMT5 (Abcam, ab109451, 1:100), anti-COLII (Thermo Fisher Scientific, MS235B, 1:100), anti-COLX (Quartett, 1-CO097-05, 1:200), anti-SOX9 (Santa Cruz Biotechnology, sc-20095, 1:50), anti-Lubricin (PRG4) (Abcam, ab28484, 1:400) and anti-RUNX2 (Medical & Biological Laboratories, D130-3, 1:100). The terminal deoxynucleotidyl transferase dUTP nick-end labeling (TUNEL) cell death assay was performed on paraffin sections with an In Situ Cell Death Detection Kit, Fluorescein (Roche) according to the manufacturer's instructions. Quantification of TUNEL-positive cells was performed on sections of three control and three mutant mice. Two to four IVDs were analyzed per mouse. Beta-galactosidase staining was performed on frozen sections of thoracic spine as previously described (Liu et al., 2015). Briefly, spines were harvested and fixed in 4% paraformaldehyde for 2 h at 4°C and decalcified with 14% EDTA at 4°C for 1 week. Tissues were washed in sucrose gradient, embedded with Tissue-Tek OCT medium, snap-frozen in liquid nitrogen and sectioned at 10 μm with a Thermo Fisher Scientific HM 550 cryostat. FISH analyses using digoxygenin-labeled antisense riboprobes for *Mmp13*, *Ihh* and *Bmp4* were performed on 5 μm paraffin sections of thoracic spine as described previously with modifications (Karner et al., 2015), and detected with a tyramine-amplified fluorescent antibody (Perkin Elmer, NEL753001KT).

### RNA isolation and real-time RT-PCR

Intervertebral discs and adjacent growth plates from the thoracic spine of the 2.5-month-old *ATC;Prmt5^f/f^* and control mice (Dox induced from 4 weeks) were isolated in cold PBS, pooled together, snap frozen and pulverized in liquid nitrogen. Three control and three mutant mice were used in each group. Total RNA was isolated using TRlzol Reagent (Invitrogen, 15596026) and cleaned up with a Direct-zol RNA miniprep kit (Zymo Research, Z2070). Reverse transcription was performed using 500 ng total RNA with an iScript cDNA synthesis kit (Bio-Rad). Real-time RT-PCR analyses were performed as previously described (Liu et al., 2015). Gene expression was normalized to β-actin mRNA and relative expression was calculated using the 2^−(ΔΔCt)^ method. Primer sequences are listed in Table S1.

## Supplementary Material

Supplementary information
