# Supplementary material for: Regulation of terminal hypertrophic chondrocyte differentiation in Prmt5 mutant mice modeling infantile idiopathic scoliosis
Source: Dis Model Mech. 2019 Dec 17;12(12):dmm041251. doi: 10.1242/dmm.041251 (PMC6955203; doi:10.1242/dmm.041251)
Supplement: Supplementary information [file dmm-12-041251-s1.pdf]

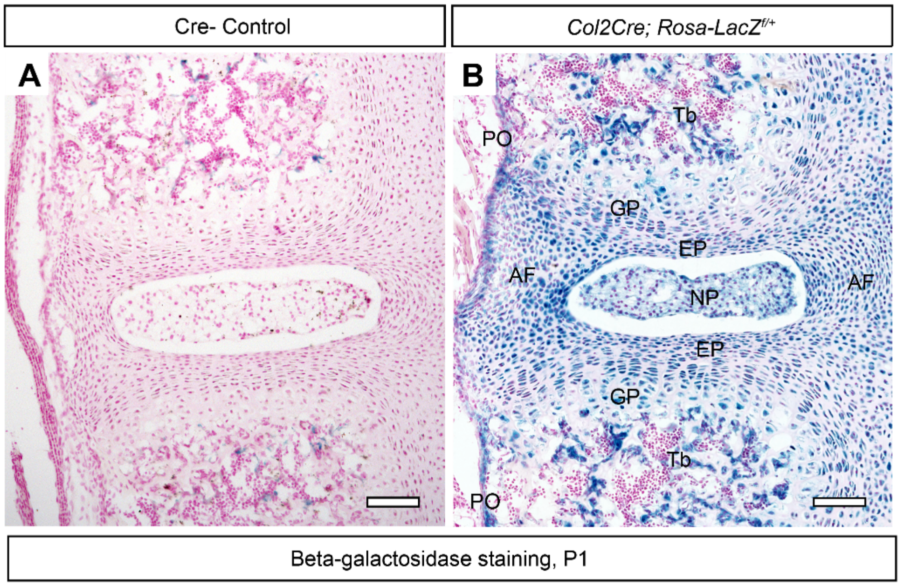

Fig. S1. The *Col2Cre* transgene targets osteochondral progenitors. (A, B) Beta-galactosidase staining on thoracic spine sections of Cre- control (A) or *Col2Cre; Rosa-LacZ*$^{f/+}$ mice (B) at P1. At this point, recombination signals (blue) can be observed in the IVD tissues, including nucleus purposes (NP), endplate (EP), and annulus fibrosus (AF), as well as the vertebral growth plate (GP), periosteum (PO), and some newly formed trabecular bone (Tb) tissues in the vertebra. (*n*=2 for each group.) Scale bar: 100μm.

Disease Models & Mechanisms • Supplementary information

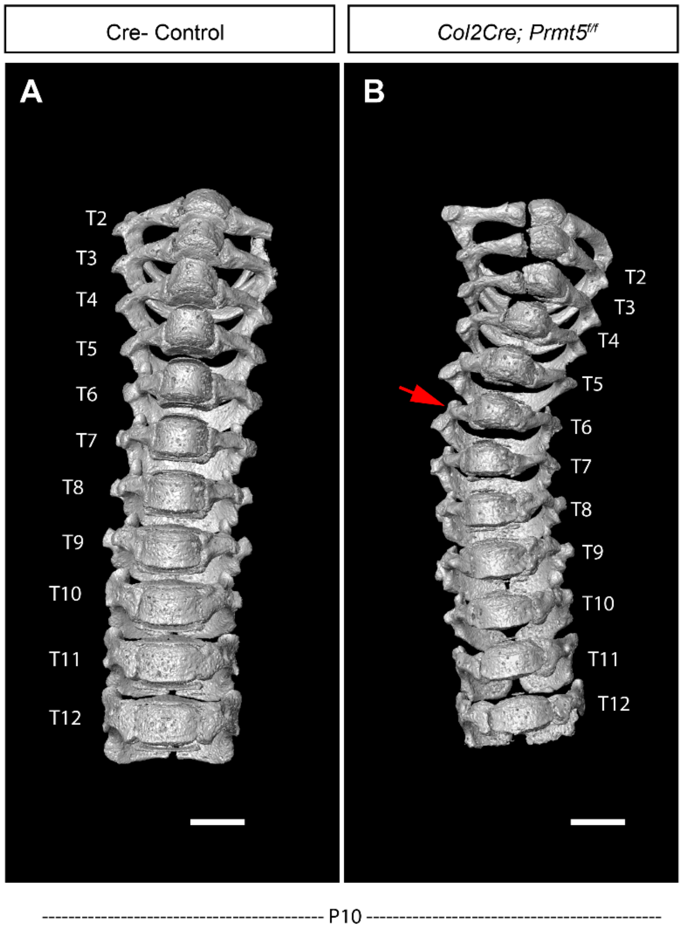

Fig. S2. MicroCT analysis shows spinal curvature in the *Prmt5* mutant mice.
(A, B) MicroCT scanning of thoracic region of the spine (T2-T12) shows IS-like curvature in the mutant mouse (B). Smaller vertebrae and a mildly wedged vertebral body at the apex of the curvature (T6) (red arrow; B) is also observed in the mutant mouse (B) compared with the control mouse (A) at P10. Scale bar: 1mm.

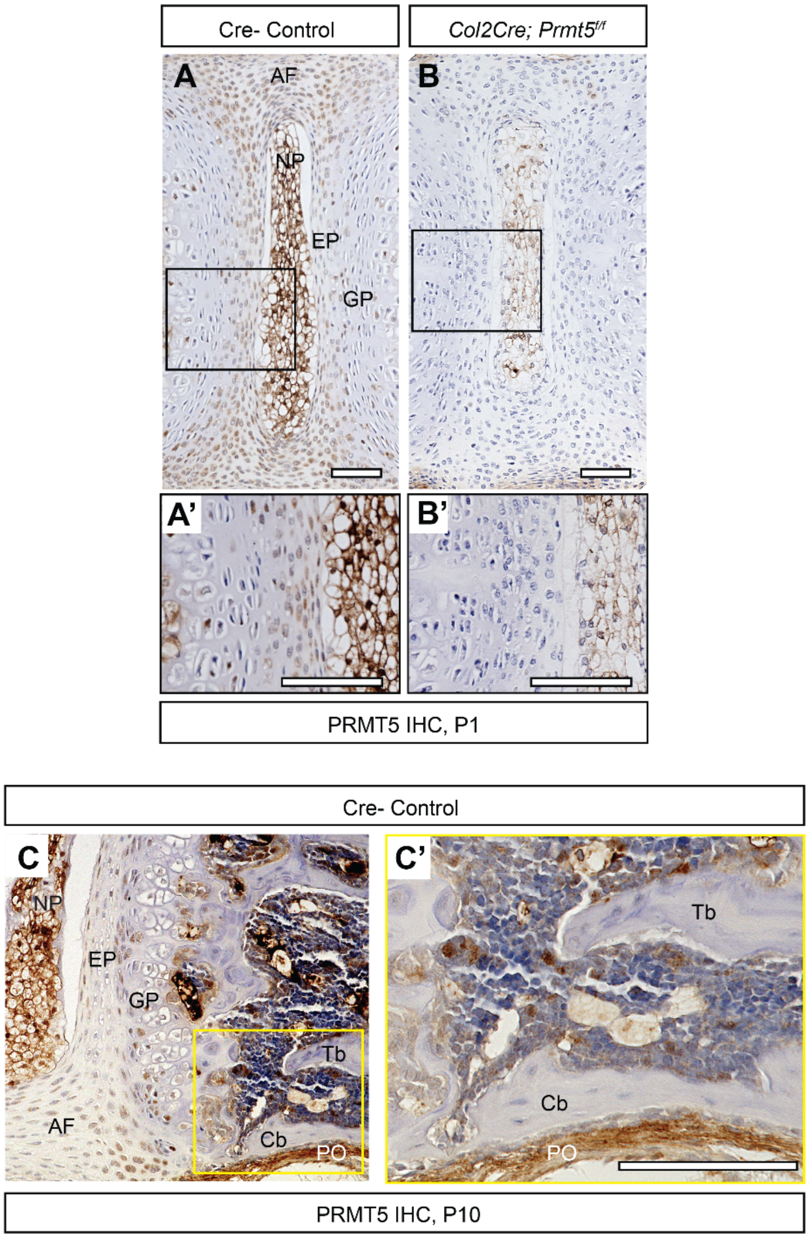

Fig. S3. PRMT5 antibody staining on spine sections of perinatal mice. (A, B) Immunohistochemistry (IHC) analysis of PRMT5 on thoracic spine sections of both control (A) and mutant (B) mice at P1. Images with higher magnification were indicated with black boxes and shown in A', B'. (n=3 for each group.) **(C)** IHC analysis of PRMT5 on thoracic spine sections of control mice at P10. Images with higher magnification were indicated with a yellow box and shown in C', demonstrating a lack of signal in the trabecular bone or cortical bone in the control mice (*n*=3 for each group). Scale bar: 100µm. *AF: annulus fibrosus, EP: endplate, NP: nucleus purposes, GP: growth plate, Tb: trabecular bone, Cb: cortical bone, PO: periosteum.*

Disease Models & Mechanisms • Supplementary information

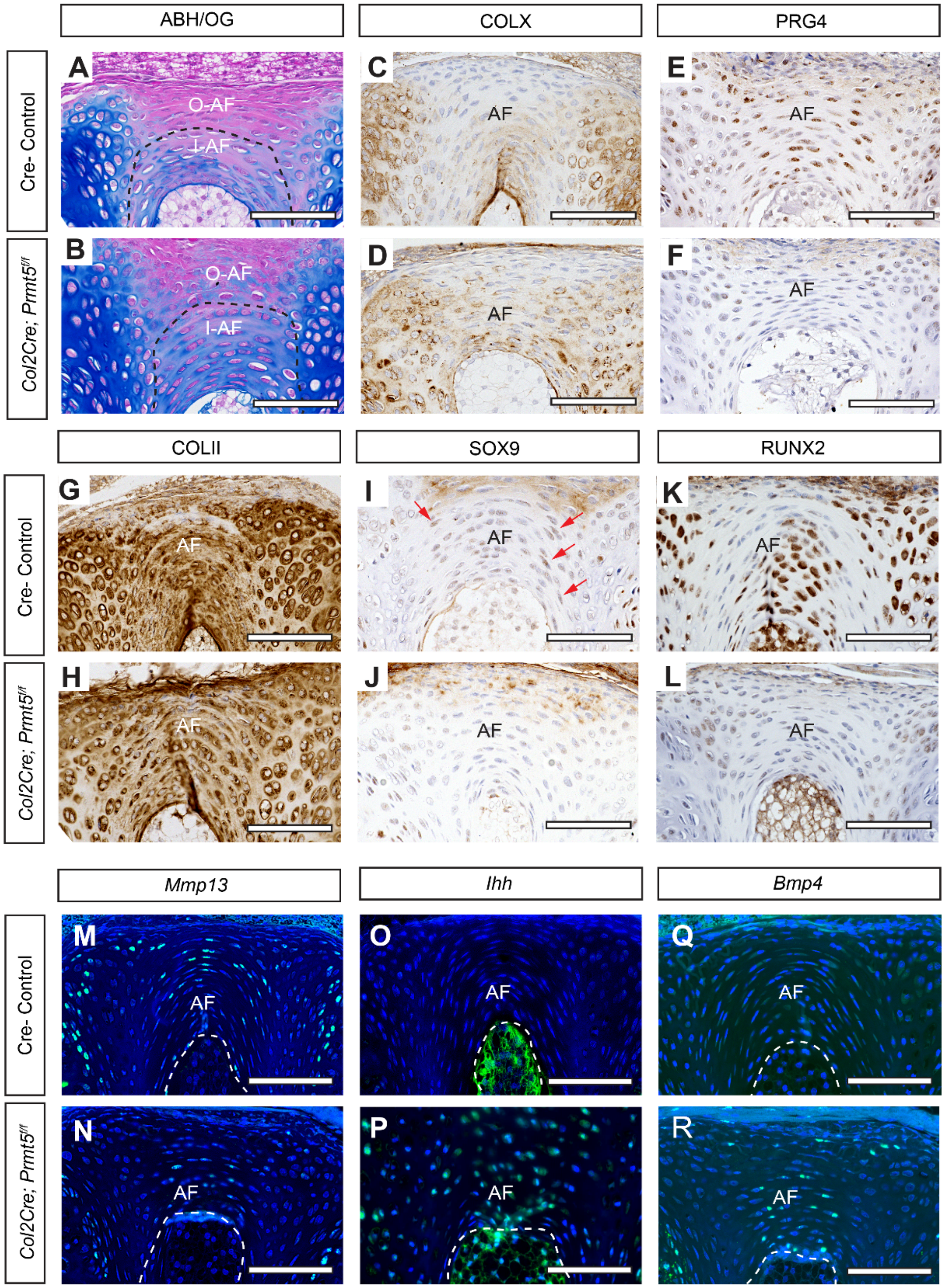

Fig. S4. Loss of *Prmt5* in osteochondral progenitor lineages of the spine results in abnormally expressed genes and proteins in the annulus fibrosus. (A, B) Alcian Blue Hematoxylin/ Orange G (ABH/OG) staining on thoracic spine sections of Cre- control (A) or *Col2Cre;Prmt5$^{f/f}$* mutant (B) mice at P10. Increased Alcian blue staining can be observed in both inner layer (I-AF) and the outer layer (O-AF) of the annulus fibrosus in the mutant mice. **(C-L)** IHC analyses of type X collagen (COLX) (C, D), PRG4 (E, F), type II collagen (COLII) (G, H),

Disease Models & Mechanisms • Supplementary information

SOX9 (I, J), and RUNX2 (K, L) on thoracic spine sections of Cre- control (C, E, G, I, K) or *Col2Cre;Prmt5$^{f/f}$* mutant (D, F, H, J, L) mice at P10. Dramatic reduction of PRG4 and RUNX2 expression are observed in the mutant annulus fibrosus (F, L) compared with the controls (E, K). Low level expression of SOX9 can be observed in the control mice (red arrows, I), however it was diminished in the mutant mice (J). No obvious difference of COLII and COLX expression can be observed in the mutant mice (D, H) compared with the controls (C, G). **(M-R)** Fluorescent *in situ* hybridization (FISH) analysis of *Mmp13*, *Ihh*, and *Bmp4* expression on thoracic spine sections of Cre- controls (M, O, Q) or *Col2Cre;Prmt5$^{f/f}$* mutant (N, P, R) mice at P10. Reduced *Mmp13* expression and ectopic *Ihh* and *Bmp4* expression is observed in the mutant annulus fibrosus (N, P, R) compared with the controls (M, O, Q). (*n*=3 for each group.) Scale bars: 100µm. *AF: annulus fibrosus; I-AF: inner layer of the annulus fibrosus; O-AF: outer layer of the annulus fibrosus.*

Disease Models & Mechanisms • Supplementary information

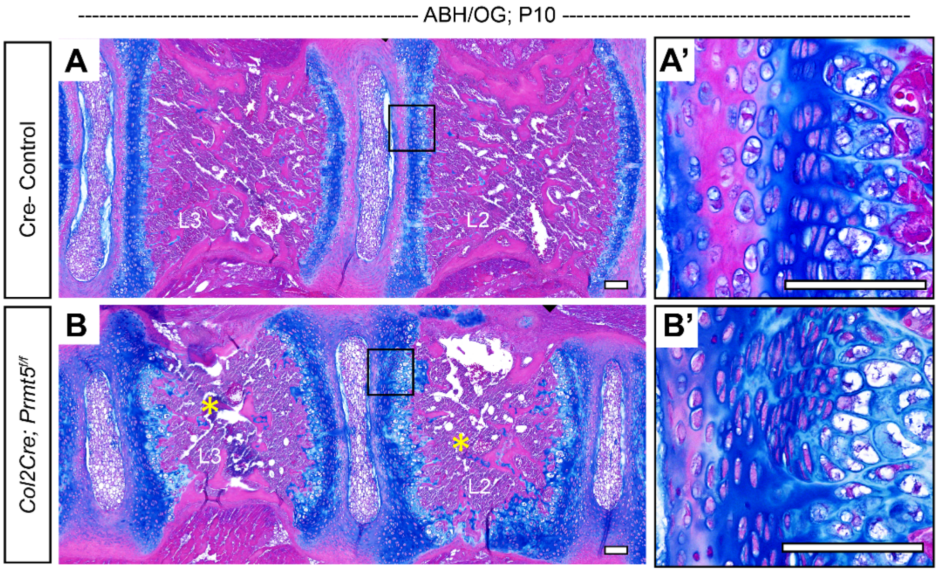

Fig. S5. Loss of *Prmt5* in osteochondral progenitor lineages of the spine results in defective ossification of the vertebral body in the lumbar region of the spine. (A-B) Alcian Blue Hematoxylin/ Orange G (ABH/OG) staining on lumbar spine sections of Cre- control (A) or *Col2Cre;Prmt5$^{f/f}$* mutant (B) mice at P10. Images with higher magnification are indicated with black boxes and shown in (A', B'). Impaired ossification in *Col2Cre;Prmt5$^{f/f}$* mutant vertebrae (yellow asterisks, B) and persistent hypertrophic growth plate (B') can be observed in mutant mice. The regions of the lumbar spine checked (L2, L3) are labeled on the vertebrae. (*n*=3 for each group.) Scale bars: 100µm.

Disease Models & Mechanisms • Supplementary information

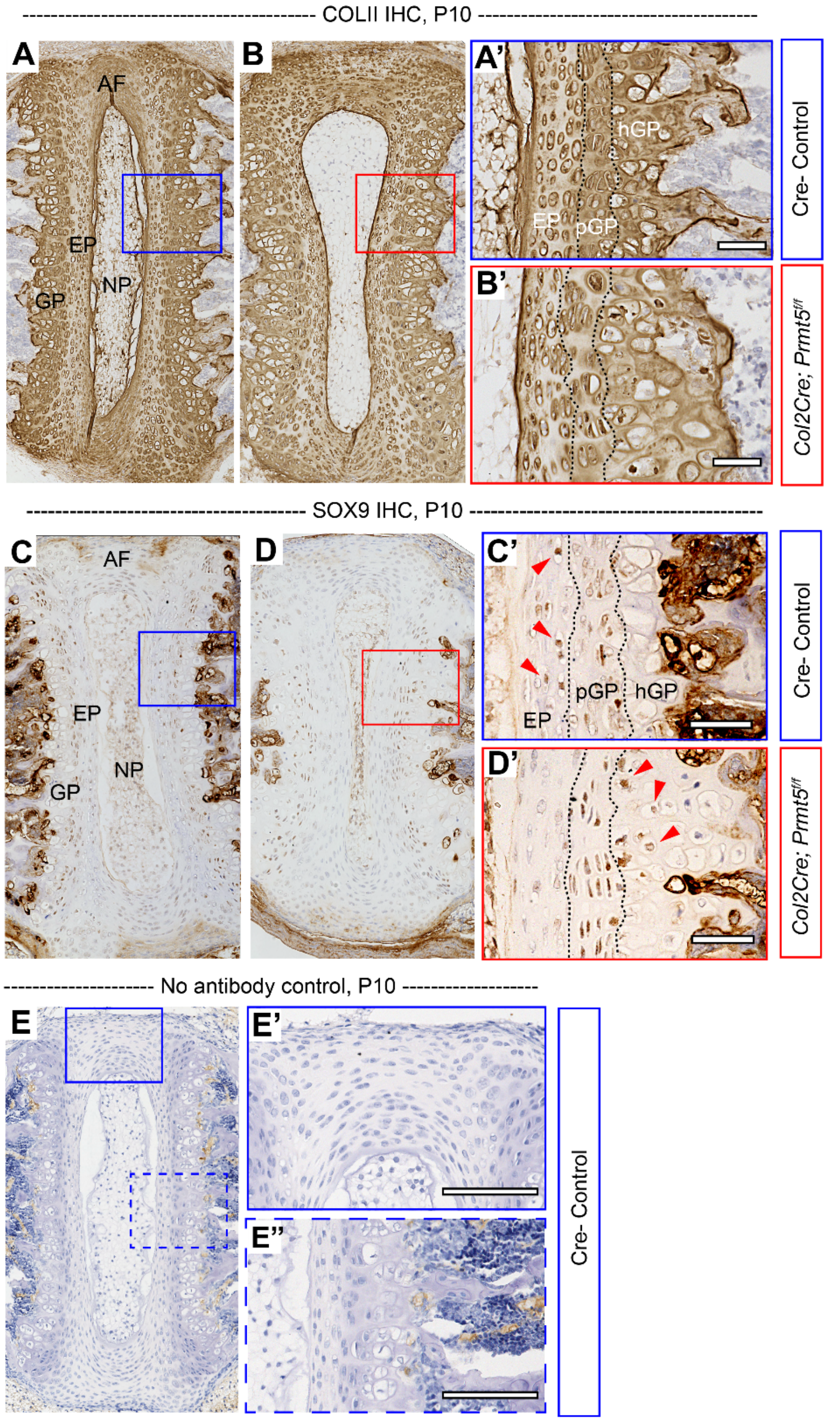

Fig. S6. Loss of *Prmt5* in osteochondral progenitor lineages of the spine results in mild reduction of COLII expression and abnormally expressed SOX9. (A-D) IHC analysis of type II collagen (COLII) (A-B') on thoracic spine sections of Cre- control (A, A') or *Col2Cre;Prmt5*$^{f/f}$ mutant (B, B') mice at P10 demonstrating typical COLII expression in mutant mice. IHC analysis of SOX9 on thoracic spine sections of Cre-control (C) or *Col2Cre;Prmt5*$^{f/f}$ mutant (D) mice at P10. Images with higher magnification were shown in (A'-D'). SOX9 is normally

Disease Models & Mechanisms • Supplementary information

expressed in the endplate (red arrowheads, C') and proliferative zone of the growth plate (C') in the control mice, but is reduced in the endplate of mutant mice (D'). Some cells in the hypertrophic zone of growth plate of mutant mice also express SOX9 (red arrowheads, D'). (*n*=3 for each group.) **(E)** No antibody control staining of the IHC analysis. Scale bars: 100µm. *AF: annulus fibrosus, EP: endplate, NP: nucleus pulposus, GP: growth plate, pGP: proliferative growth plate, hGP: hypertrophic growth plate.*

Disease Models & Mechanisms • Supplementary information

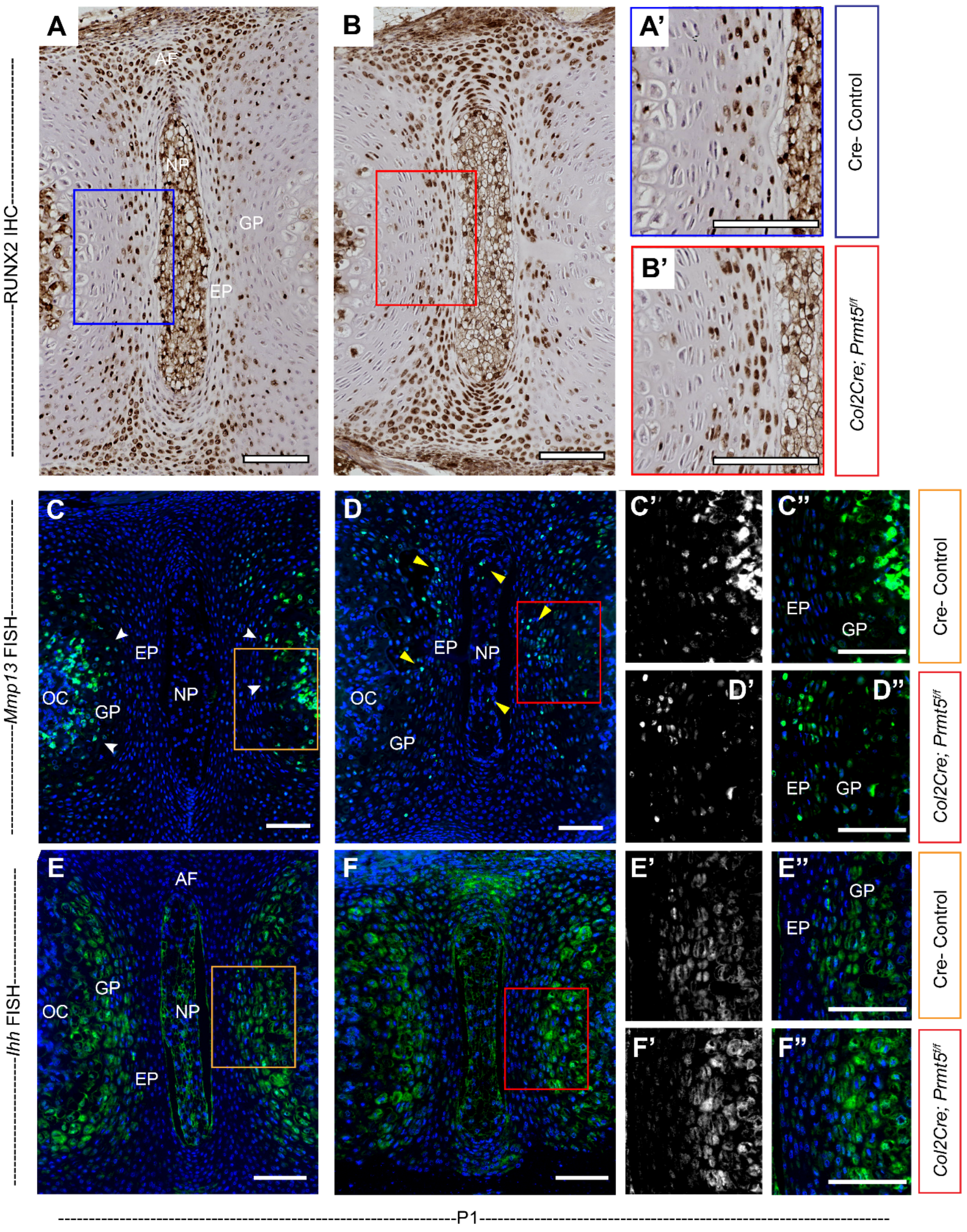

Fig. S7. Loss of *Prmt5* in osteochondral progenitor lineages of the spine results in regularly expressed RUNX2 and *Ihh* but abnormally expressed *Mmp13* in newborn mice. (A, B) IHC analysis of RUNX2 on thoracic spine sections of Cre- control (E) or *Col2Cre;Prmt5*$^{f/f}$ mutant (F) mice at P1 demonstrating typical RUNX2 expression in mutant mice. Images with higher magnification were shown in (A’, B’). **(C, D)** Fluorescent *in situ* hybridization (FISH) analysis

with DAPI (nuclei) counterstain (blue) of *Mmp13* on thoracic spine sections of Cre- control (A) or *Col2Cre;Prmt5$^{f/f}$* mutant (B) mice at P1. Strong *Mmp13* signal was detected in the vertebral growth plate and the newly formed ossification center in the control spine (white arrowheads; C). However, it was almost depleted in the growth plate and the OC in the mutant mice (D). On the contrary, ectopic *Mmp13* signal was observed in the endplate and the nucleus purposes in the mutant mice (yellow arrowheads; D). (C', D') are greyscale *Mmp13* fluorescent *in situ* channels, and (C, C", D, D") are merged channels. Images with higher magnification were shown in (C', C" and D', D"). **(E, F)** FISH analysis of *Ihh* on thoracic spine sections with DAPI (nuclei) counterstain (blue) of Cre- control (A) or *Col2Cre;Prmt5$^{f/f}$* mutant (B) mice at P1. Comparable expression pattern of *Ihh* was detected in both control and mutant mice. (E', F') are greyscale *Ihh* fluorescent *in situ* channels, and (E, E", F, F") are merged channels. Images with higher magnification were shown in (E', E" and F', F"). (*n*=3 for each group.) Scale bars: 100µm. *OC: ossification center, NP: nucleus purposes, EP: endplate, GP: growth plate.*

Disease Models & Mechanisms • Supplementary information

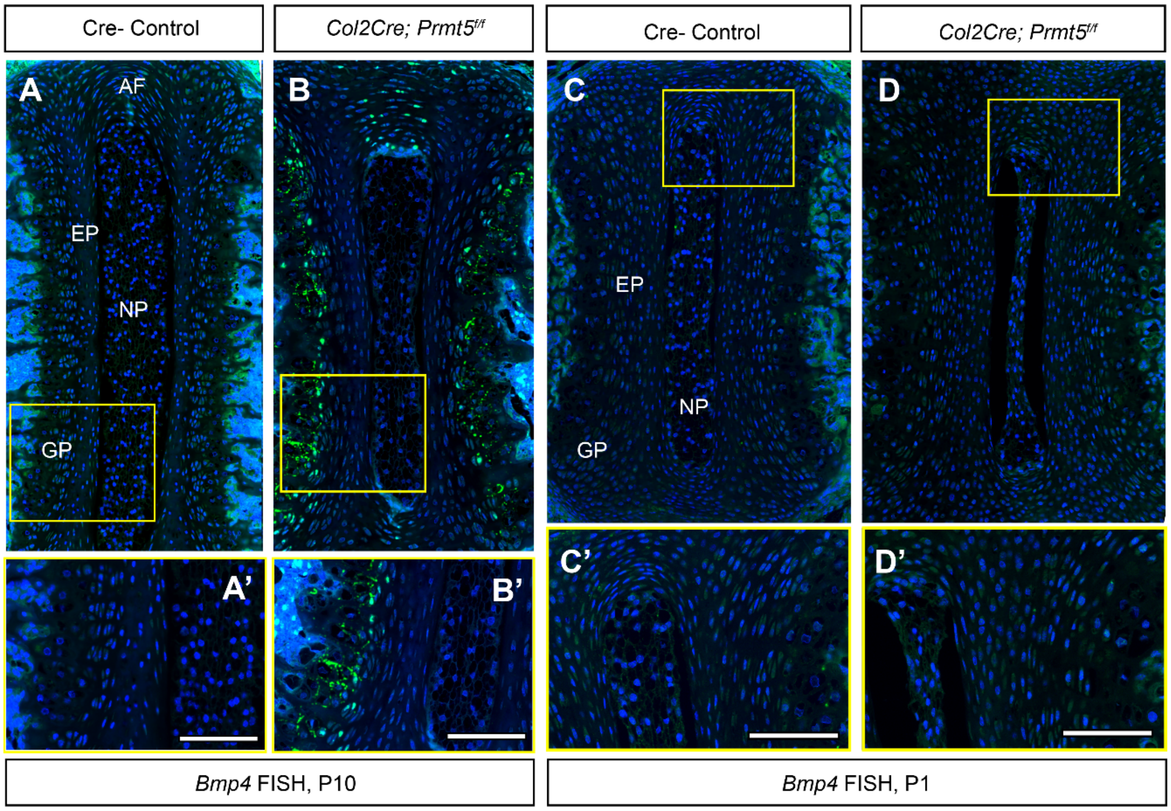

Fig. S8. Loss of *Prmt5* in osteochondral progenitor lineages of the spine induces *Bmp4* expression at P10, but not at P1. (A-D) FISH analysis demonstrating *Bmp4* expression on thoracic spine sections of Cre- controls (A and C) or *Col2Cre;Prmt5$^{f/f}$* mutant (B and D) mice. Images with higher magnification were indicated with yellow boxes and shown in A'-D'. At P10 we observe increased *Bmp4* expression in annulus fibrosus of the IVD and in the vertebral growth plate (B, B') compared to an absence of expression in control mice (A, A'). However, at P1 this *Bmp4* signal was not detected in either genotype. (*n*=3 for each group.) Scale bars: 100µm. *AF: annulus fibrosus, NP: nucleus purposes, EP: endplate, GP: growth plate.*

Disease Models & Mechanisms • Supplementary information

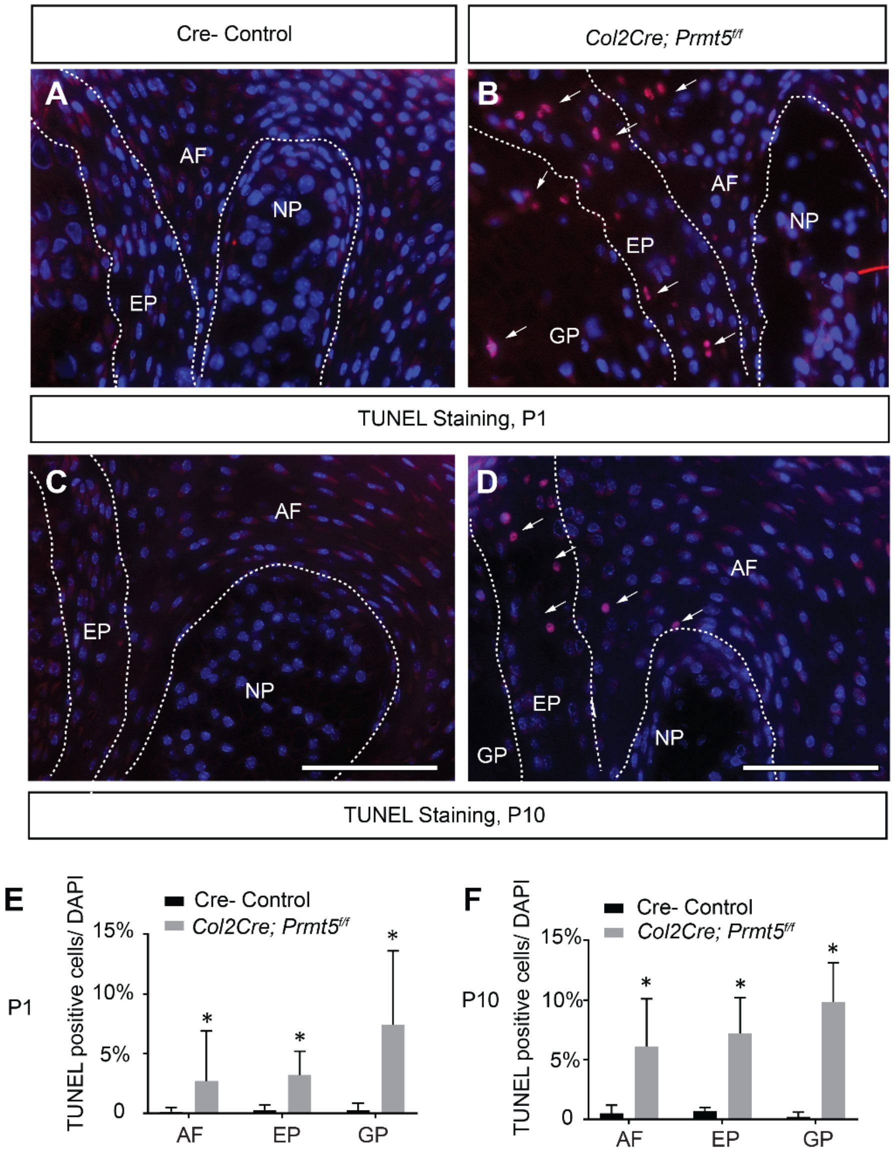

Fig. S9. Loss of *Prmt5* in osteochondral progenitor lineages of the spine results in increased cell death in the intervertebral disc and the growth plate. (A-D) TUNEL staining on thoracic spine sections of Cre-control (A, C) or *Col2Cre;Prmt5$^{f/f}$* mutant (B, D) mice at P1 (A, B) and P10 (C, D). TUNEL positive cells were indicated with white arrows. **(E, F)** Quantification of TUNEL positive cells to DAPI positive cells in the cartilaginous tissues of the spine at P1 (E) and P10 (F). "*" indicates $p<0.05$, two-tailed student t-test. (*n*=3 for each group.) Scale bars: 100µm in (A-D). *AF: annulus fibrosus, EP: endplate, NP: nucleus pulposus, GP: growth plate.*

Disease Models & Mechanisms • Supplementary information

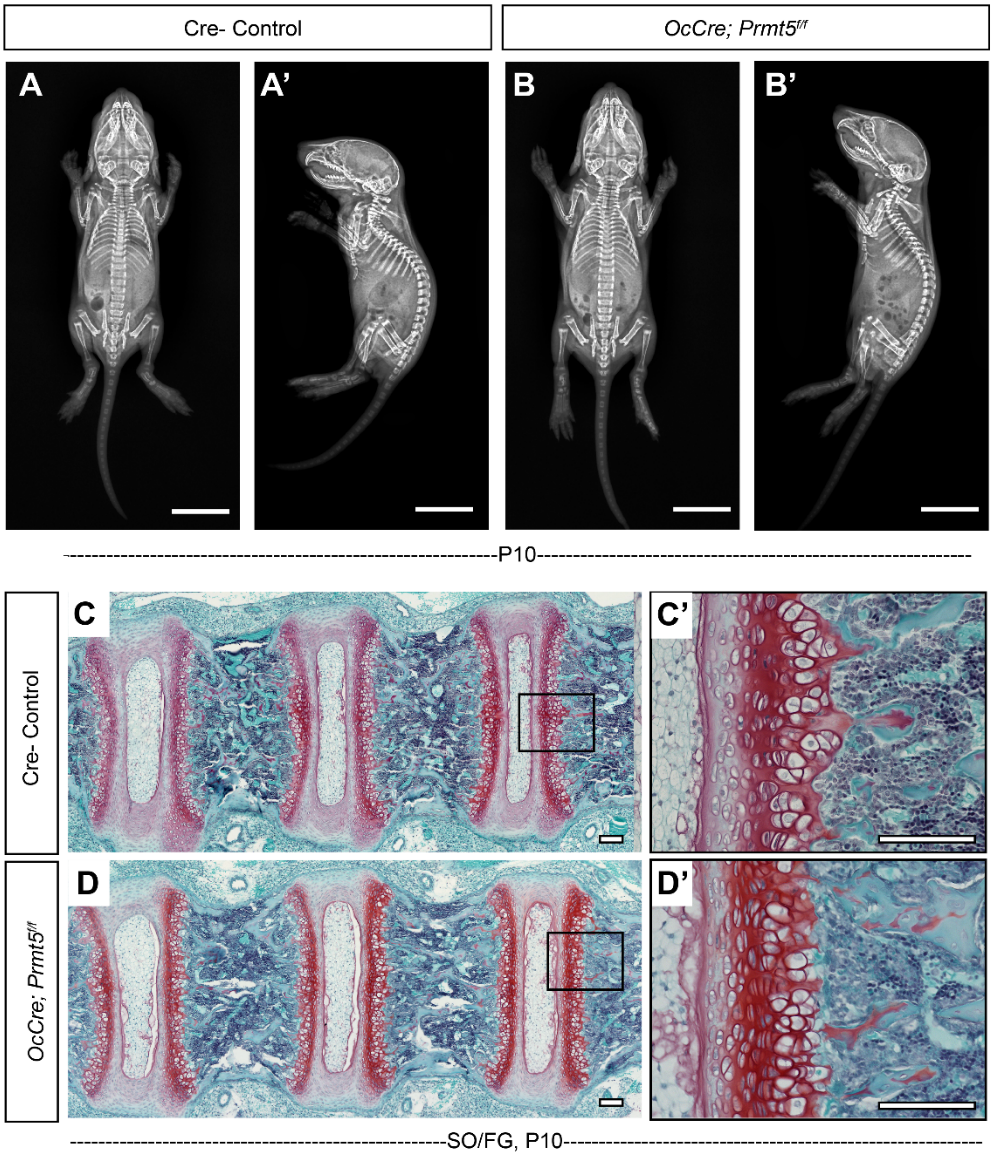

Fig. S10. No scoliosis or spine deformity was observed in the *OcCre; Prmt5$^{f/f}$* mice. (A, B) X-ray imaging analysis showed no scoliosis in neither Cre- control (A, A') nor *OcCre; Prmt5$^{f/f}$* mutant (B, B') mice at P10. **(C, D)** Safranin O/Fast Green (SO/FG) staining on thoracic spine sections of Cre- control (C, C') or *OcCre; Prmt5$^{f/f}$* mutant mouse at P10. Images with higher magnification are indicated with black boxes and shown in C' and D'. (*n*=4 for each group.) Scale bars: 10mm in (A-B'), 100μm in (C-D').

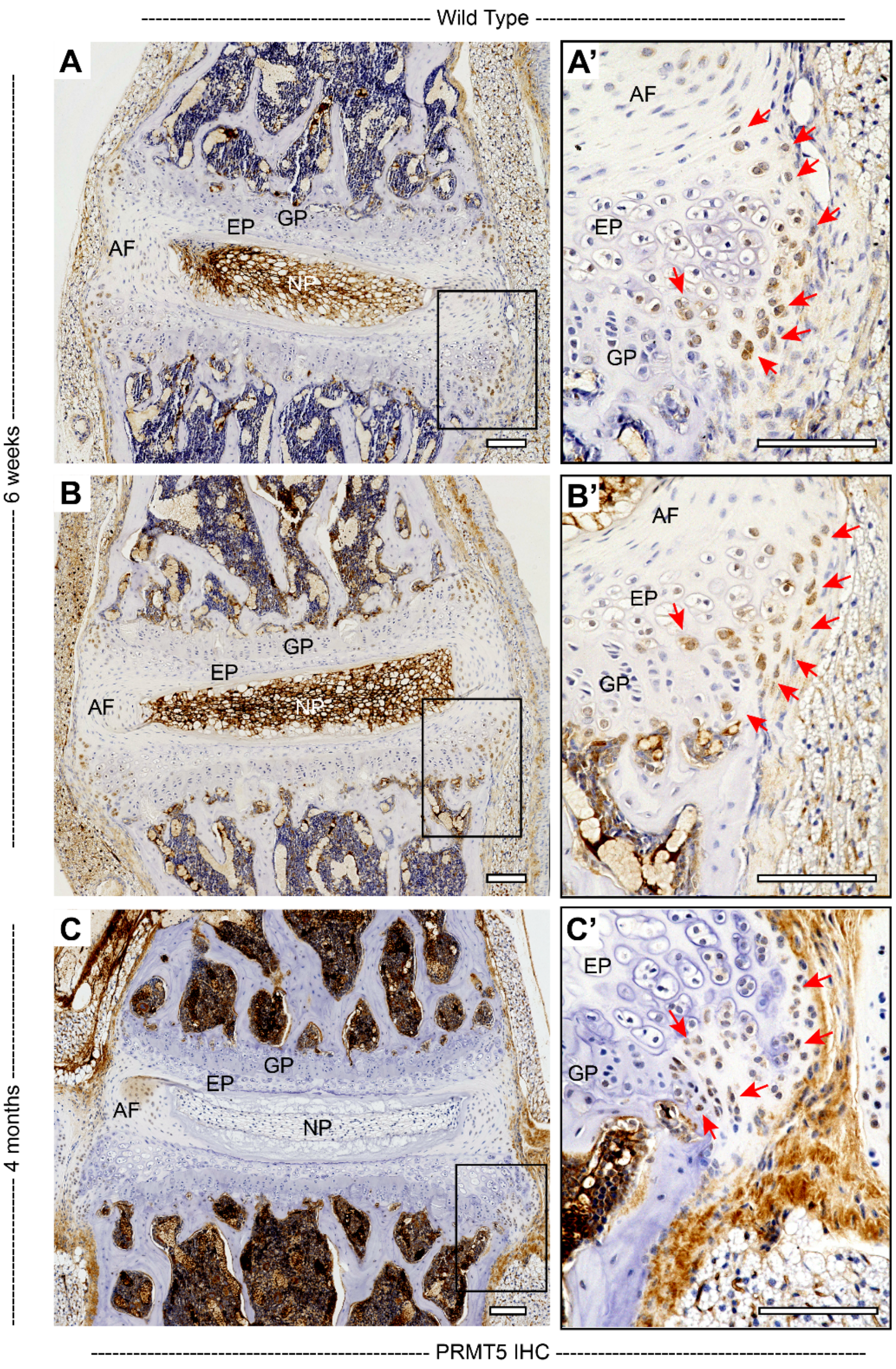

Fig. S11. Low-level expression of PRMT5 was detected in postnatal spine. (A-C) Immunohistochemistry (IHC) analysis of PRMT5 on thoracic spine sections of wild type mice at 6-weeks of age (A-B’) or 4-months of age (C, C’), respectively. Images with higher magnification were indicated with black boxes and shown in A’-C’. Red arrows indicate PRMT5 positive cells. Scale bars: 100µm. *AF: annulus fibrosus, EP: endplate, NP: nucleus purposes, GP: growth plate.* (*n*=3 for each group.)

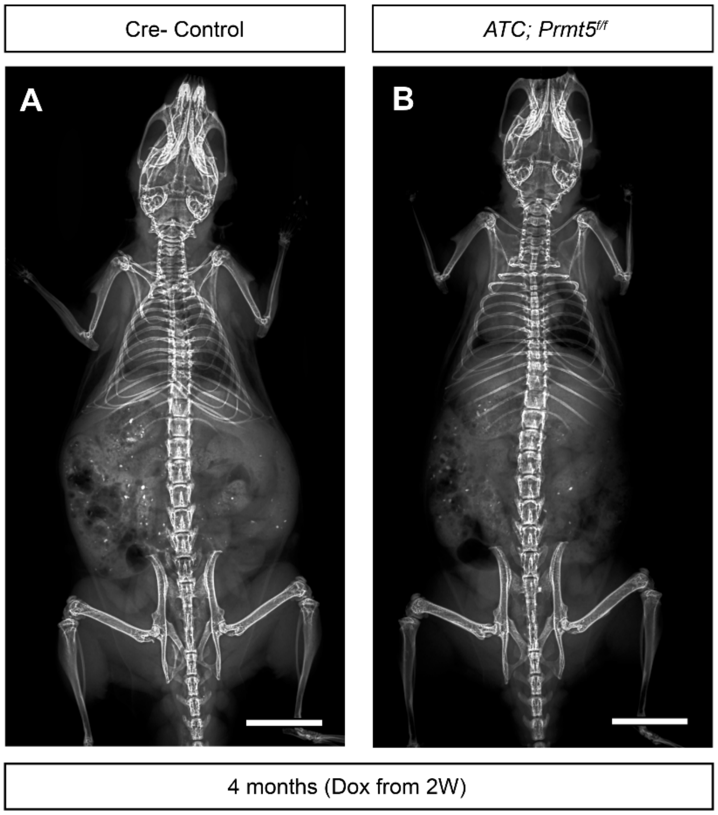

Fig. S12. No scoliosis was observed in the *ATC; Prmt5$^{f/f}$* mice when induced at 2-weeks of age. (A, B) X-ray imaging analysis showed no scoliosis in neither Cre- control (A) nor *ATC; Prmt5$^{f/f}$* mutant (B) mice at 4-months of age (Dox induced from 2 weeks). (*n*=3 for each group.) Scale bars: 1mm.

Table S1. qPCR primer used in this study.

Gene ID	Forward primer (5'-3')	Reversed primer (5'-3')
b-actin	AGATGTGGATCAGCAAGCAG	GCGCAAGTTAGGTTTTGTCA
Prmt5	CTGAATTGCGTCCCCGAAATA	AGGTTCCTGAATGAACTCCCT
Sox9	AGGAAGCTGGCAGACCAGTA	CGTTCTTCACCGACTTCCTC
Col2a1	ACTGGTAAGTGGGGCAAGAC	CCACACCAAATTCCTGTTCA
Acan	CGTGTTTCCAAGGAAAAGGA	TGTGCTGATCAAAGTCCAG
Prg4	AAACAGCCAATAAGAGCCCTTGGC	TGGCTTTGACTTGGCTTTGACACG
Col10a1	CTTTGTGTGCCTTTCAATCG	GTGAGGTACAGCCTACCAGTTTT
Mmp13	AGACTGGTAATGGCATCAAGG	GCCATTTCATGCTTCCTGATG

Disease Models & Mechanisms • Supplementary information

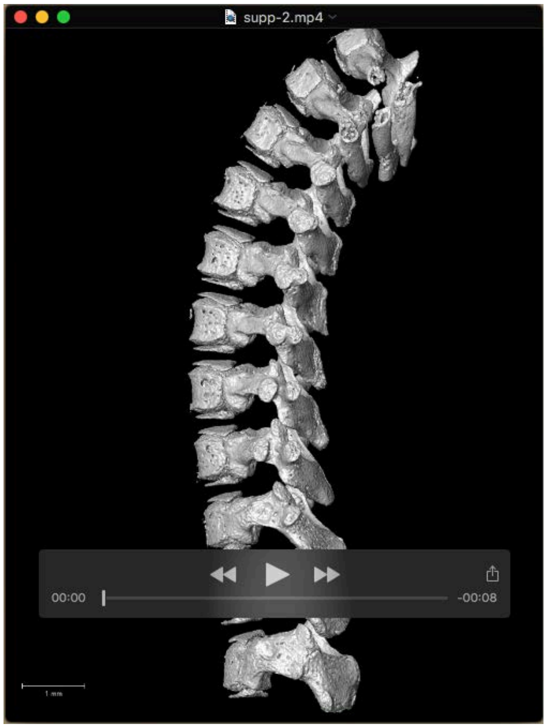

Movie 1. Three dimensional rotation view of the thoracic spine of Cre- control mouse at P10.

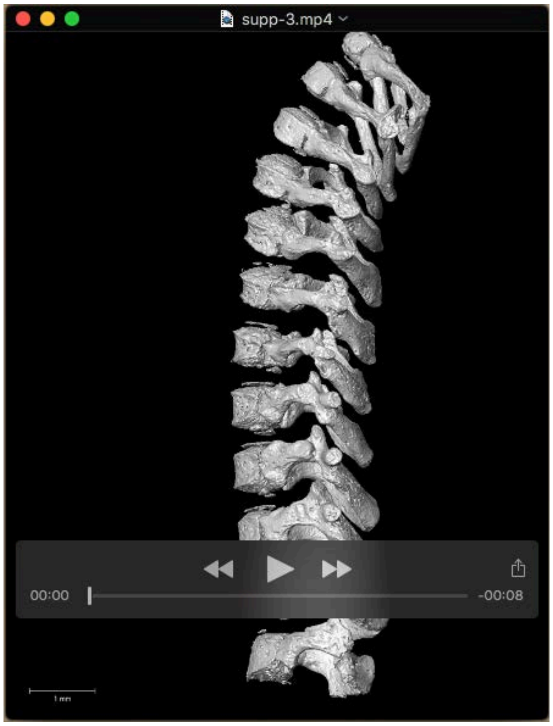

Movie 2. Three dimensional rotation view of the thoracic spine of *Col2Cre; Prmt5$^{f/f}$* mutant mouse at P10.
